# Performance Analysis of Packet Aggregation Mechanisms and Their Applications in Access (e.g., IoT, 4G/5G), Core, and Data Centre Networks †

**DOI:** 10.3390/s21113898

**Published:** 2021-06-04

**Authors:** Godlove Suila Kuaban, Tülin Atmaca, Amira Kamli, Tadeusz Czachórski, Piotr Czekalski

**Affiliations:** 1 Institute of Theoretical and Applied Informatics, Polish Academy of Sciences, Bałtycka 5, 44-100 Gliwice, Poland; gskuaban@iitis.pl; 2 Faculty of Automatic Control, Electronics and Computer Science, Department of Distributed Systems and Informatic Devices, Silesian University of Technology, Akademicka 16, 44-100 Gliwice, Poland; piotr.czekalski@polsl.pl; 3 Samovar, Télécom Sud Paris, Institut Polytechnique de Paris, 9, rue Charles Fourier, 91011 Evry, France; atmaca.tulin@gmail.com (T.A.); amira.kamlistag@gmail.com (A.K.)

**Keywords:** packet aggregation, Internet of Things (IoT), programming Protocol-independent Packet Processor (P4), performance evaluations, mobile networks (4G/5G), optical backbone/metro networks, diffusion approximation

## Abstract

The transmission of massive amounts of small packets generated by access networks through high-speed Internet core networks to other access networks or cloud computing data centres has introduced several challenges such as poor throughput, underutilisation of network resources, and higher energy consumption. Therefore, it is essential to develop strategies to deal with these challenges. One of them is to aggregate smaller packets into a larger payload packet, and these groups of aggregated packets will share the same header, hence increasing throughput, improved resource utilisation, and reduction in energy consumption. This paper presents a review of packet aggregation applications in access networks (e.g., IoT and 4G/5G mobile networks), optical core networks, and cloud computing data centre networks. Then we propose new analytical models based on diffusion approximation for the evaluation of the performance of packet aggregation mechanisms. We demonstrate the use of measured traffic from real networks to evaluate the performance of packet aggregation mechanisms analytically. The use of diffusion approximation allows us to consider time-dependent queueing models with general interarrival and service time distributions. Therefore these models are more general than others presented till now.

## 1. Introduction

The amount of traffic generated by various access networks such as Digital Subscriber Lines (DSLs), ethernet Local Area Networks (LANs), wireless LANs, mobile networks (e.g., 3G, 4G, and 5G networks), and recently, the Internet of Things (IoT) networks is increasing exponentially, and have likely increased significantly since the beginning of the year 2020. The recent increase in the amount of traffic carried over the Internet could be attributed to the global reaction to the outbreak of the COVID-19 pandemic by transferring some key services (e.g., health care consultation, education, retail, entertainment, and business and work-related activities), and the recent increase in the rate of adoption of IoT. The packet sizes generated from these access networks vary from a few bytes in IoT and wireless sensor networks to 1500 bytes in Internet Protocol (IP) networks. The transmission of massive amounts of smaller packets, broadband access networks and high-speed core networks introduces some challenges such as bandwidth wastage due to protocol overhead, inefficient use of network resources, and increased energy consumption. It is, therefore, essential to develop strategies to deal with the huge amounts of traffic generated by access networks, especially the IoT network [1]. One of the strategies to increase bandwidth efficiency, ensure efficient use of network resources, and reduce the extra energy consumed due to the presence of huge amounts of small packets is to incorporate packet aggregation modules in some nodes of the network. Despite its benefits, packet aggregation increases the packets’ delays [2] and may not be suitable for traffic belonging to real-time applications [3].

The industry 4.0 trend is transforming every industry’s production capabilities, including health care, energy, agriculture, food chains, logistics, retail, transportation and manufacturing, with IoT low power connectivity as its driving force [4]. IoT devices are designed to minimise their energy consumption and hence increase their battery life. The energy consumption in IoT devices is usually lowered by reducing its computing power by using microcontrollers or microprocessors, minimising their storage capacity, reducing the amount of energy consumed during communication with the use of low power communication protocols, and implementing energy-efficient encryption schemes and security protocols. The low power communication protocols such as Constraint Application Protocol (CoAP) [5], Message Queueing Telemetry Transport (MQTT) [6], Advanced Message Queueing Protocol (AMQP) [7], and Light Weight Machine-to-Machine (LWM2M) [8] communication protocols that have been proposed are designed to deminish energy consumption by reducing the size of the IoT packet payload. The time required to receive or transmit a packet depends largely on its size, which is directly correlated with the amount of energy required to receive or transmit the packet. It implies that the smaller the packet’s size, the smaller the communication duration, the smaller the amount of energy required to receive or transmit the packet, and hence, the longer the battery lifetime. Therefore, low power communication protocols are designed to keep the packet size as small as possible. However, with the recent large scale proliferation of IoT sensor devices that generate massive amounts of relatively small packets, it is necessary to think about the various ways that packet aggregation schemes can be deployed to deal with the challenges introduced at the level of access networks, Internet core networks, and data centres.

The recent generations of mobile networks (e.g., 4G and 5G networks) are designed to support IoT devices and satisfy the requirements of various IoT applications. However, mobile networks were initially designed to support user equipment (e.g., mobile phones, tablets), generating traffic where packet sizes are larger than those from IoT devices. The transmission of IoT traffic directly over mobile networks results in the inefficient utilisation of radio resources. The amount of IoT data carried over mobile networks is expected to increase significantly and poses challenges for service providers [9]. To ensure efficient utilisation of radio resource for IoT over mobile network deployment, a packet aggregation scheme can be implemented at an intermediate node between the IoT devices and the radio access network [10]. In this case, multiple IoT packets are aggregated into a larger packet whose size is almost equivalent to the maximum packet size that can be transmitted over the mobile network to efficiently utilise the radio resources. Instead of wasting resource blocks to handle individual small IoT packets, the IoT packets are aggregated, and the aggregated IoT packets share only one resource block. Moreover, the sizes of the packets from the user equipment in mobile networks (e.g., 4G and 5G) are smaller than the IP packet sizes handled in its transport networks. The aggregation and multiplexing of fronthaul and backhaul traffic into IP packets before transmission in the transport network of 5G Cloud Radio Access Network (C-RAN) was discussed in [11,12,13].

Similarly, the packets from access networks that are transported over optical networks are smaller than standardised optical packets. Transporting smaller packets from the access networks directly over the optical network results in bandwidth wastage due to protocol overhead and poor utilisation of network resources. Moreover, at every node, the optical packet is converted from the optical domain to the electrical domain to perform routing and regenerates the signals and is then converted back to the optical domain for transportation; this is the so-called electronic bottleneck [14], that increases the energy consumption. The electronic packets from the access networks are aggregated into larger optical packets to ensure efficient bandwidth and resource utilisation in the optical core networks. The optical packets are transported purely in the optical domain through the optical core network without being converted to the electrical domain. They are transported transparently from the ingress edge nodes to the egress edge nodes and boosted using optical amplifiers when the signal power falls below acceptable limits.

The Maximum-Time (MT), the Maximum-Size (MS), and the Maximum-Time-Maximum-Size (MTMS) packet aggregation schemes are the most popular packet aggregation schemes that have been implemented in commercial network equipment. Recently, MT, MS, and MTMS packet aggregation schemes have been implemented in the programming Protocol-independent Packet Processor (P4) [15] hardware switches in [3,16,17,18], to exploit its programmability, hardware speed, and flexibility. In the time-based or size-based, or hybrid (a combination of time threshold and size threshold criteria) packet aggregation scheme, when packets arrive at the network node, they are classified based on their destination, Class of Service (CoS) or Quality of Service (QoS) parameters, and queued in the input buffer. The smaller packets stored in the input buffer are aggregated into larger ones when the number of these packets stored in the buffer is greater than or equal to a defined maximum value or when a defined time threshold is reached. The main drawback of these packet aggregation mechanism is that in low traffic applications like in the case of IoT, the maximum defined size threshold may rarely be reached, resulting in excessive delays [16], which could be mitigated by setting the maximum time threshold to be within the maximum delay tolerance. Moreover, the MT packet aggregation scheme may result in large variation in the aggregated packet sizes; hence, poor resource utilisation at the level of the core network [19]. A novel slot-based packet aggregation scheme was recently proposed in [20,21]. In this mechanism, the small packets stored at the input buffers are aggregated into larger ones and scheduled for transmissions during preallocated time slots.

The performance evaluation of time-based, size-based, or hybrid packet aggregation schemes at the edge node of IP over all-optical networks have been presented in [22,23,24,25,26,27]. The major drawback of these studies is the assumption that the interarrival times of IP packets into the aggregation buffer follows a Poisson distribution, which is far from reality, as it differs significantly from the measured interarrival times from the Center for Applied Internet Data Analysis (CAIDA) repository which we used in [28,29]. The authors in [18] used the Poisson assumption to analyse the performance of a time and size based packet aggregation scheme for IoT traffic over Software Defined Network, but the measured interarrival times for IoT traffic reported in [30] significantly differs from Poisson distribution. Diffusion approximation-based performance evaluations models which use measured interarrival times and sizes of packets measured from real networks have been proposed in [27,31], and this paper is an extension of these works. Diffusion approximation is a well-established modelling approach used to study non-Markovian queueing systems in which the arrival times and service times distributions are general, which was proposed in the current form by Gelenbe in [32,33].

This paper presents a review of packet aggregation applications in access networks (IoT and 4G/5G mobile networks), optical core networks, and cloud computing data centre networks. We also propose analytical models for the evaluation of the performance of packet aggregation mechanisms. We demonstrate the use of measured traffic from real networks to evaluate analytically the performance of packet aggregation mechanisms. The rest of the paper is organized as follows: In Section 2, we present a review of the applications of packet aggregation mechanisms in access, core, and data centre networks. We present a comparison between theoretical and measured traffic models used to model the performance of computer and telecommunication networks. We present a methodology to evaluate the performance of a time-based and sized-based packet aggregation mechanism in Section 4 and then present another methodology for the evaluation of the performance of a slot-based packet aggregation mechanism in Section 5. Section 4 and Section 5 present the original contributions of the paper. We conclude the paper in Section 6.

## 2. A Review of Recent Applications of Packet Aggregation in Computer and Telecommunication Networks

**Figure 1:**
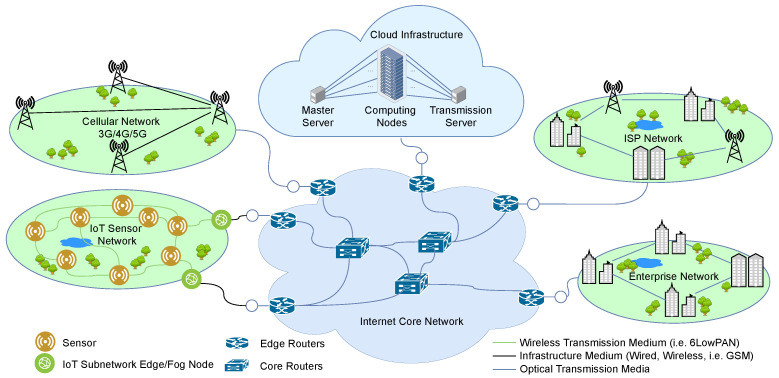
An architectural model consisting of access networks, Internet core networks and the cloud data centre.

This section presents a review of recent applications of packet aggregation in computer and telecommunication networks, from the access networks (e.g., IoT, wireless sensor, 4G/5G mobile networks), through Internet core networks to cloud data centre networks. We present in Figure 1 an abstract architecture of a network in which the IoT and wireless sensor networks, the cellular networks (3G/4G/5G), the Internet Service Provider (ISP) access networks, enterprise access networks, and data centre networks are connected by a high-speed internet core network. Many papers have been published on the application of packet aggregation to improve throughput efficiency, improve resource utilization, and reduce energy consumption in computer networks, but we will limit our review to recent works.

### 2.1. Packet Aggregation in IoT and Wireless Sensor Networks

A simplified architecture for IoT applications consists of the IoT layer, the fog layer (if fog computing is supported), and the cloud layer. The IoT sensors measure relevant data from the environment, securely transfer the data through an access point to the fog nodes for lightweight processing or the cloud data centre for advanced data analytics. The feedback from the data analytics platforms (fog and data centre applications) is sent back to perform appropriate actions to control some IoT actuators or to provide information to users for decision making. Well-known low power, reliable wireless access communication technologies such as LoRaWAN [34], Sigfox [35] have been widely adopted for the communication between the sensor devices and the access point. The size of the IoT packet generated is small, bringing spectral inefficiency, poor resource utilisation, and high energy consumption. Therefore, the IoT packets can be aggregated at the level of the access point or fog node (because they have higher computing resources than the IoT devices and do have energy limitations as they are continuously powered by a reliable energy source) before being transmitted through the Internet core network to the cloud computing data centres.

The authors in [36] proposed a packet aggregation scheme for the aggregation of IoT packets in wide area networks. In their scheme, when packets arrive at the access point, they are classified based on their destination, and packets belonging to non-real-time applications are aggregated, but those that belong to delay-sensitive real-time applications are transmitted immediately to their destination. A “flag” field is added to the packets. It is checked by every node to determine whether the packet should be aggregated (if it does not belong to a real-time application) or not. They assumed that not every node in the network should possess the ability to aggregate and disaggregate packets, which should be considered when choosing the next node during packet forwarding. A similar dynamic mechanism for the aggregation of packets in IoT and Low power and Lossy Networks (LLNs) to decrease energy consumption and increase battery life was proposed in [37]. In the proposed scheme, each node is equipped with a learning automaton [38], which grants permission to the node to aggregate small packets that need to be aggregated into a larger one, and denies aggregation permission for some packets that need to be transmitted immediately.

In the packet aggregation scheme proposed in [39] to reduce delays and energy consumption in body sensor networks, the IoT packets are stored in local buffers and aggregated into larger ones before forwarding them. When the number of packets stored in the buffer is greater or equal to the packet aggregation threshold or when the first packet’s waiting time is greater than the waiting time aggregation threshold, the stored packets are aggregated into a larger one and sent to the forwarder node. The forwarder node is selected based on the expected queue size and waiting time to ensure the acceptable quality of service.

In the packet aggregation schemes proposed in [36,37,39], the authors classified the packets into packets that belong to real-time applications and those that belong to non-real-time applications. However, the authors did not clarify whether the both kinds of packets share the same buffer or not. If they share the same buffer, then the QoS experienced by packets that belong to real-time applications may be degraded as the packet aggregation mechanism may introduce additional delays. The authors in [40] proposed priority-based channel access and data aggregation scheme to reduce packet delays in clustered Industrial IoT networks. When the IoT packets arrive at the access point or fog node, they are classified into high priority (that is, packets that belong to real-time applications) and low priority packets (those that belong to non-real-time applications). The high priority packets are stored in a high priority queue and transmitted without aggregation, but the low priority packets are stored in a low priority queue and aggregated into larger ones before transmission.

### 2.2. Packet Aggregation in IoT over SDN-Based Network Networks

The packet aggregation and disaggregation mechanisms implemented in most traditional network devices (servers, switches and routers) are performed by the Central Processing Unit (CPU), which execute software programs to perform packet aggregation and packet disaggregation operations in the control plane. The throughput rates achieved with CPU-based packet aggregation and disaggregation operations are lower than those obtained by using hardware ASIC switches. It is because software execution speeds are lower than those of hardware switching ASICs [3]. The authors in [3,16,17,18] have demonstrated an SDN approach in which the data plane pipelines of P4 hardware switches can be programmed to perform packet aggregation and disaggregation operations at high speed. The authors conducted experiments using IoT traffic, which makes P4 SDN-based data plane switches a good choice for deploying networks that carry IoT traffic.

The recent introduction of the software defined networking paradigm has given network operators and service providers programmatic control over the networking equipment in their networks’ data planes. The development of P4 technology has provided hardware leverage for manufacturers of network equipment and network operators. The P4 language is a high-level programming language used to program the data plane of hardware switches based on the SDN networking paradigm. It is used to program hardware switches similarly to Verilog, and VHDL (Very high-speed Hardware Description Language) hardware description languages are used to program FPGA(Field Programmable Gate Arrays)-based hardware. However, the P4 language does not require a detailed understanding of the underlying electronic design as in the case with VHDL, and Verilog [17]. Therefore, the ability to programmatically manipulate packet payload in P4 switches enables flexible and faster (higher throughputs) packet aggregation and disaggregation operations than those obtained when using traditional (non-programable) network devices.

**Figure 2:**
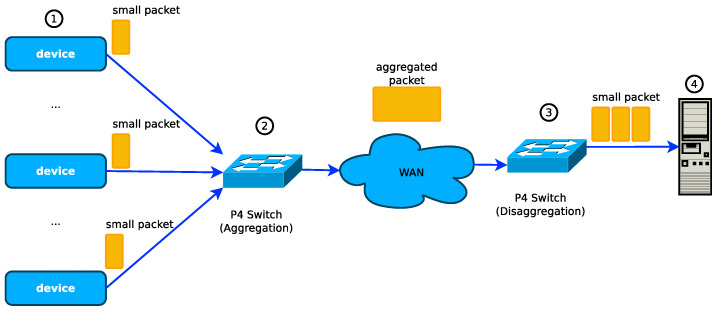
Architecture of a demonstration of IoT packet aggregation and disagreggation using P4 switches [16].

The implementation of packet aggregation of IoT traffic on P4 switches was first proposed in [18] as shown in Figure 2. The authors discussed its feasibility, presented performance evaluations models to evaluate the performance of packet aggregation and disaggregation operations. The first design and implementation of packet aggregation and disaggregation in a P4 switch was presented in [3]. In their implementation, the packet aggregation and disaggregation operations are performed purely in the hardware switching ASIC pipelines, and the authors achieved a 100 Gbps packet aggregation throughput which is the highest so far reported. The limitation of these studies is that their implementation could only aggregate fixed-sized IoT packets, but we may have traffic from different sources with different packet sizes in a real network. The authors addressed this limitation in their recent implementation in [16], where they designed and implemented packet aggregation and packet disaggregation operations in P4 SDN data plane switches that enable the aggregation and disaggregation of IoT packets of different payload sizes with a throughput of 100 Gbps. Recently, the authors in [17] proposed a P4 implementation of an IoT protocol designed to ensure an adaptable aggregation of packets to reduce the number of packets sent over the network with an acceptable delay.

### 2.3. Packet Aggregation in IoT over Mobile Networks (4G/5G)

With the widespread adoption of Low Power Wide Area (LPWA) technologies to enable long-range communication for IoT devices, NB-IoT (NarrowBand-IoT) [41] has been introduced and can coexist with existing mobile networks (e.g., 2G/3G/4G/5G). Existing mobile networks may be overwhelmed in the future by the massive growth in IoT traffic [10] when hundreds of billions of IoT devices are connected to the Internet via mobile networks. Allocating radio resources for each IoT packet will result in spectral inefficiency and inefficient radio resources utilisation. Multiple small IoT packets can be aggregated into larger ones so that a group of aggregated IoT packets can share the same radio resource.

The authors in [10] proposed introducing a Relay Node (RN) between the IoT devices and the 5G radio access network. It receives small IoT packets, stores them in a buffer and then aggregates them into larger packets that are transmitted to the cellular radio access network through a wireless connection. It improves improves spectral efficiency and radio resource utilisation, but it also introduces a significant delay. This is why packets from real-time IoT applications should not be aggregated. A similar approach was proposed in [9] for LTE-A(Long Term Evolution Advanced) mobile network in which an intermediate node is placed between the IoT devices and the 4G radio access network. It stores temporally IoT packets, and aggregate them when the queue size of packets in the buffer reaches a defined maximum threshold or when a defined waiting time threshold is reached. The authors concluded that their approach guarantees enhanced spectral efficiency, increases the capacity of the radio access network, and ensures an acceptable delay. The drawback of the approach proposed in [9,10] is that both real-time and non-real-time traffic share the same buffer, which causes real-time traffic to suffer from excessive delays due to the packet aggregation. Real-time packets should be placed in higher priority queues and relayed directly to the radio access network without aggregation, or traffic from real-time IoT applications should be transmitted directly to the radio access network.

### 2.4. Packet Aggregation in 5G Radio Access Networks (C-RAN)

**Figure 3:**
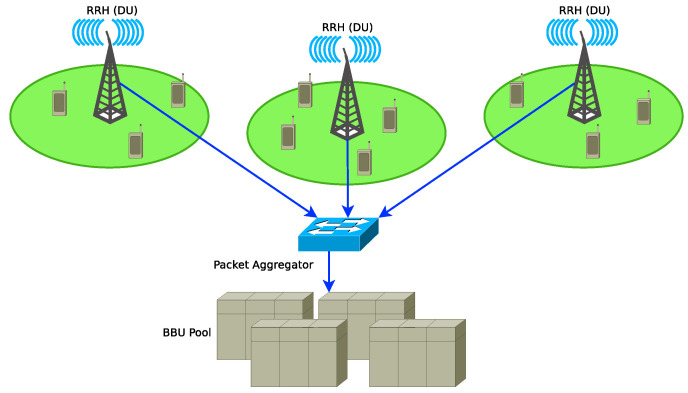
A Cloud Radio Access Network (C-RAN) architecture with packet aggregation at the fronthaul network [11].

A 5G Cloud Radio Access Network (C-RAN) consists of a set of Remote Radio Heads (RRHs), a fronthaul network and a pool of shared Broadband Units (BBUs) as shown in Figure 3. The C-RAN paradigm is based on splitting functionalities by shifting complex signal processing from the RRHs to the BBUs. It leverages on the benefits provided by Network Function Virtualization (NFV) and SDN technologies to add flexibility and adaptability to fronthaul and transport networks of 5G mobile networks [12]. The RRHs receives the radio signals, digitise them and then transmit them to a pool of BBUs through the fronthaul network. The packets that belong to the flows coming from the RRHs are smaller than those in the backhaul network (packets from fronthaul networks are smaller than IP packets in the backhaul networks). An ethernet switch is deployed to aggregate packet flows from the fronthaul network and then multiplex them with those from the backhaul networks and transported through optical links to a pool of BBUs.

The authors in [11] proposed a C-RAN architecture shown in Figure 3 in which an ethernet switch connected to the RRHs aggregates fronthaul traffic and forward the aggregated traffic to the cloud. The authors in [12] discussed the problem of multiplexing and aggregating fronthaul and backhaul traffic on C-RAN optical ethernet link. A strategy to aggregate fronthaul packet frames to improve throughput efficiency of the transport network of a 5G cloud radio access network was discussed in [13]. The analysis of the delay introduced by packet aggregation in 5G C-RAN has not been discussed so far. It will require the modelling of the packet aggregation process and considering the characteristics of 5G traffic and packet sizes.

### 2.5. Packet Aggregation in IP over All-Optical Networks

**Figure 4:**
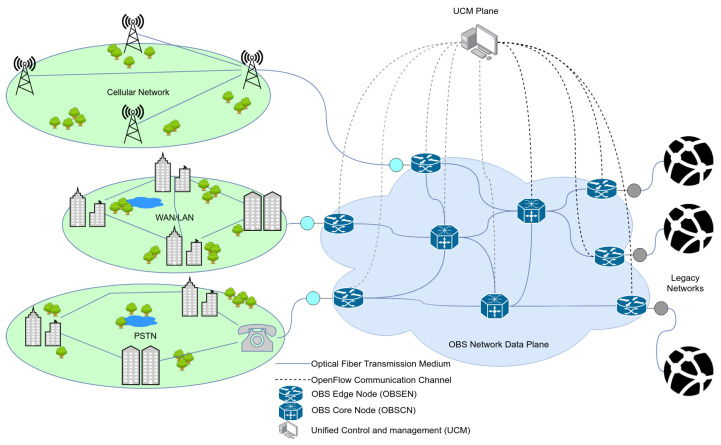
An architecture of an OBS network.

The continuous rapid growth in the traffic generated by access networks and transported over long haul transport networks have led to the development and deployment of high-speed all-optical transport core networks. Small packets from the access network need to be aggregated into larger ones which are converted into optical packets and transported transparently across the optical transport networks. Due to the challenges in developing optical switches with optical memory, Optical Burst Switching (OBS) networks architectures (e.g., see Figure 4) have been adopted as networking solution for optical networks.

The process of packet aggregation/disaggregation in optical networks have been discussed in [22,23,24,25,26,42,43,44].

### 2.6. Packet Aggregation in Cloud Computing Data Centre Networks

Cloud computing is a well-known dynamic, cost-effective and scalable computing paradigm that enables on-demand remote access of computing resources such as software, infrastructure, and platform over the internet. The large scale adoption of cloud computing is due to the introduction of virtualization technology which makes cloud computing scalable. Virtualization refers to the hardware or software methods that enable the partitioning of a physical machine into multiple instances that run concurrently and share the underlying physical resources, and devices. A Virtual machine monitor (VMM), also called a hypervisor, is used to manage the VMs and enable them to share the underlying physical resources including the network devices [45]. Some of the tools that enable the deployment of virtualization in cloud computing data centres include KVM, UMLinux, VMware, VirtualBox and Xen [46].

**Figure 5:**
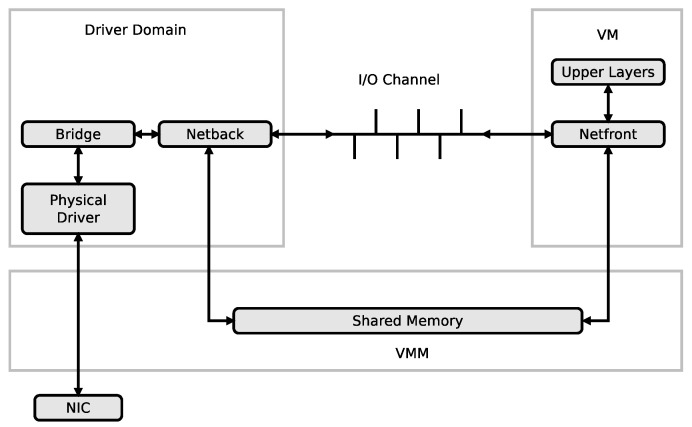
Driver domain based I/O virtualization [47].

In a virtualization environment like in the Xen environment, the driver domain hosts the physical device drivers, and network virtualisation is therefore essential to provide connectivity between the driver domain and the virtual machines (VMs) as seen in Figure 5. The I/O channel that transfers packets between the driver domain and the virtual machines creates a bottleneck due to its poor throughput [46,47]. Packet aggregation has been proposed as a strategy to increase the communication throughput between the driver domain and the virtual machines by 700% in [45,46,47]. The packets to be transfered from the driver domain to the virtual machines are classified based on their destination Mac addresses, aggregated and placed in containers which are transfered through the I/O channel to the virtual machines. At the virtual machine domain, the packets are dissggregated back into the small packets and relayed to the upper layers for processing. After processing, the packets are aggregated again, placed into containers and send back to the driver domain through the same channel.

## 3. Traffic Models: Theoretical and Measured Traffic Models

To design and evaluate the performance of packet aggregation algorithms, network equipment designers and network operators often use discrete event simulation and mathematical modelling. Mathematical models make it possible to develop mathematical relationships between the design parameters of the algorithm and its performance parameters. The limitation of most of the proposed mathematical models for packet aggregation algorithms (e.g., [22,23,24,25,26,42,43,44]) is that they are based on the assumption that the distribution of the interarrival times of packets into the buffer follows a Poisson distribution. It is often assumed that the distribution of the packet sizes is fixed or exponentially distributed.

**Figure 6:**
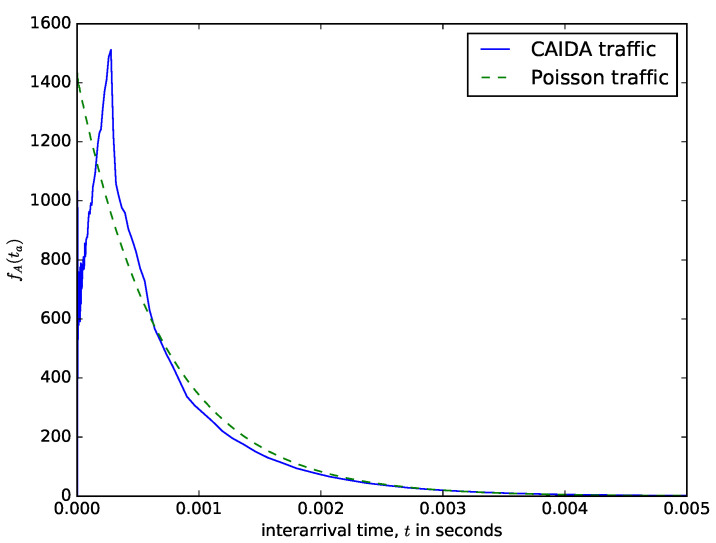
Interarrival time distribution $f_{A}\left(t_{a}\right)$, from the CAIDA measurement of the Equinix Chicago link compared with the exponantial distribution with the same mean.

**Figure 7:**
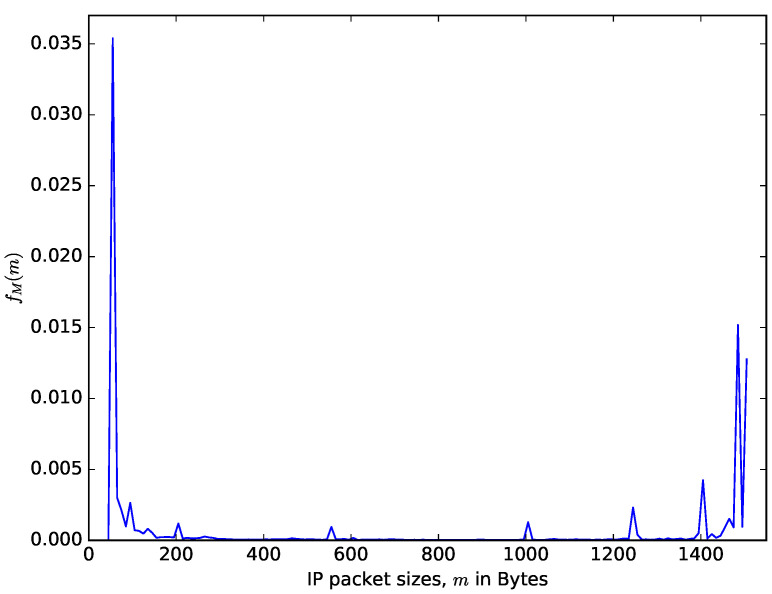
Distribution of the IP packet sizes, $f_{M}\left(m\right)$ from the CAIDA measurement of the Equinix Chicago link.

Figure 6 shows a comparison of theoretical distribution of the interarrival times of packet based on the Poisson arrival process assumption and the distribution of measured interarrival times from the CAIDA traffic data repository [48]. CAIDA routinely collects traces on chosen backbone links and make them available for research purposes. The data sets contain timestamps provided with up to nanosecond precision but truncated and stored in pcap (traffic capture) format with microsecond timescale. They also provide a dataset of the packet sizes. It can be observed in Figure 6 that for the values of the interarrival time that are less than 0.002 seconds, the distribution of the probability density of the interarrival time from the CAIDA data sets significantly differs from the theoretical one obtained using the Poisson assumption. However, for the values of the interarrival times that are greater than 0.002, the distributions from the CAIDA data sets and that from the Poisson traffic are similar. The authors in [49] made a statistical study of the interarrival times of measured IP traffic comparing them with Weibull, Pareto 2, Gamma, exponential, and lognormal distributions. They found the best fit for Pareto 2 distribution. Therefore, even though assuming that the interarrival times of packets follow a Poisson process facilitates performance analysis, the limitation of this assumption should be noted. Figure 7 shows the distribution of the packet sizes, with a sharp spike at about 64 bytes (for signalling packets) and another spike at about 1500 bytes (the maximum IP packet size). The average IP packet size from the presented dataset is 698 bytes. The distribution of the packet sizes is completely different from the usual assumption that it is exponentially distributed (e.g., see [24]). The datasets presented in Figure 6 and Figure 7 are datasets of IP v4 packet sizes and their interarrival times from the Equinix Chicago link collected during one hour on 18 February 2016, having 22,644,654 packets belonging to 1,174,515 IPv4 flows (see [50]).

The squared coefficient of variation of the distribution of the interarrival times of the CAIDA traffic shown in Figure 6 is 1.02, i.e., close to that of the Poisson traffic where it is 1.0. It could justify the use of the Poisson assumption. The authors in [18] evaluated the performance of a packet aggregation mechanism for IoT traffic over SDN data plane made up of P4 switches. They assumed that the distribution of the interarrival time of IoT packets into the aggregation buffer follows a Poisson process.

**Figure 8:**
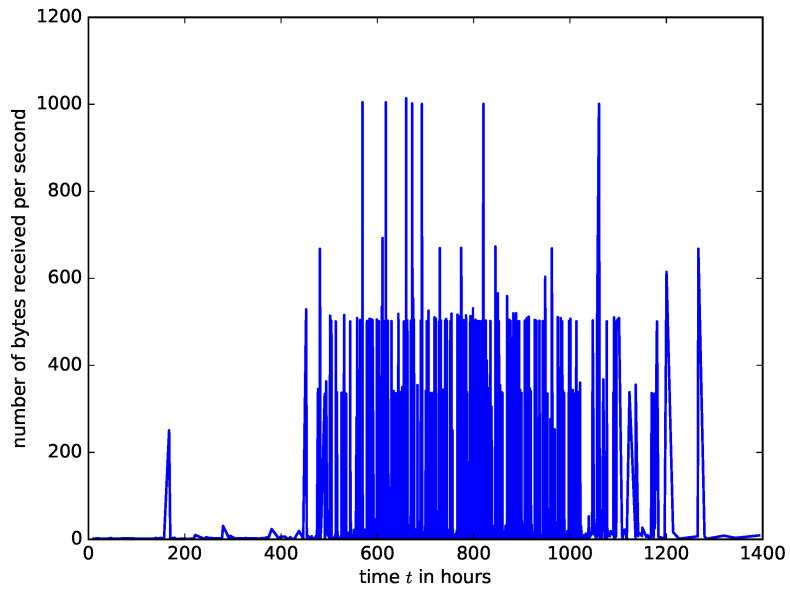
A trace of the arrival intensities of IoT traffic generated using data from a real IoT network, collected in [30].

However, the measured arrival intensities of IoT packet into a buffer in an access point are shown in Figure 8. The IoT traffic trace in Figure 8 was generated from a smart IoT environment with 28 different IoT devices such as cameras, light bulbs, motion sensors, health monitors etc. It was collected for six months [30]. We used the traffic traces from [30] to create a traffic generator of IoT traffic which was sent to a network device acting as an IoT access point. The number of bytes arriving at the buffer of the network device at one second time window was measured. It can be observed that the characteristics of IoT traffic are entirely different from that of IP traffic. The IoT traffic is made of spikes because the IoT sensors’ measurements for a given IoT application are updated at a predefined time simultaneously. At some time instants, there is heavy traffic from the sensors followed by prolonged silence. Therefore, analysing the performance of network devices carrying IoT traffic using the Poisson arrival assumption will yield inaccurate results.

It can be observed from Figure 6 and Figure 7 that the distributions of the interarrival times and packet sizes of network traffic differ from usual Poisson assumptions. Diffusion approximation may use the mean and variance taken from real traffic traces and use them to estimate the diffusion process parameters, as it was done it [27,31].

## 4. Performance Analysis of Time-Based and Size-Based Packet Aggregation Mechanisms

In this section, we present diffusion approximation models for the time-based and size-based packet aggregation mechanism.

When the first packet arrives, the byte counter and time counter are initialized. The byte counter tracks the number of bytes accumulated in the buffer, and when it is greater than or equal to the defined threshold, then the content of the buffer is aggregated into a larger packet and sent to the transmission module for transmission. The timer tracks the waiting time of the first packet in the buffer to ensure that packets do not wait for too long in the buffer during a low traffic period and when it reaches a predefined waiting time threshold, the content of the buffer is aggregated and sent to the transmission module for transmission.

We propose analytical models for the evaluation of the time-based, size-based and hybrid packet aggregation mechanism. We compare the results of the analytical models with simulations. A simulation is a technique in which a virtual environment that emulates the behaviour of a physical system is created in software. A very popular type of simulation used in the performance evaluation of computer systems and networks is called discrete event simulation. We used a discrete event simulator that was programmed using the Java programming language. The simulator consists of a traffic generator created using the CAIDA traffic data sets and a buffer which represents the buffer at the input port of the node where the aggregation is performed. The MT, MS and MTMS packet aggregation mechanism is programmed in the simulator. At each simulation run, we collect the data on the aggregated packet sizes, and the interarrival times are collected and plotted together with the distributions from the analytical models using Matplotlib (a plotting library based on the python programming language). For both modelling and simulation, we use the datasets of IP v4 packet sizes and their interarrival times from the Equinix Chicago link collected during one hour on 18 February 2016, having 22,644,654 packets belonging to 1,174,515 IPv4 flows (see [50]). The traffic parameters are: m=698 bytes, $\sigma_{M}^{2}$ = 449,361, λ=70 packets per second, $\sigma_{A}^{2}=4.9358e^{-7}$.

### 4.1. Diffusion Approximation of the Aggregation Buffer

When packets arrive in the buffer, the number of bytes in the buffer increases and the buffer content continues to grow until the maximum defined size threshold or the waiting time threshold is reached. We represent the growth process of the content of the buffer by a diffusion approximation [32,33,51] process. Suppose that a diffusion approximation process X(t) represents the number of bytes stored in the buffer at time *t*, then the dynamic changes in the number of bytes accumulated in the buffer can be modelled by diffusion equation (which is a parabolic partial differential equation describing Brownian motion of tiny particles) [50]

(1) $$\frac{\partial f(x,t;x_{0})}{\partial t}=\frac{\alpha }{2}\frac{\partial^{2}f\left(x,t;x_{0}\right)}{\partial x^{2}}-\beta \frac{\partial f(x,t;x_{0})}{\partial x}\;$$

where βdt and αdt represent the mean and variance of the changes of the diffusion process at dt. The equation defines the conditional probability density function (pdf) of the diffusion process X(t)

$$f\left(x,t;x_{0}\right)dx=P\left[x\leq X\left(t\right)<x+dx\;\;|\;\;X\left(0\right)=x_{0}\right].$$

The diffusion approximation applied to queueing systems is based on the assumption that the number of arrivals of customers joining the queue during a random time *T* has a distribution that is close to normal and does not depend on the distribution of interarrival times but only on its two first moments. The mean and variance of this normal distribution are λT and $\lambda^{3}\sigma_{A}^{2}T$ where 1/λ and $\sigma_{A}^{2}$, are mean and variance of interarrival times [32]. Here, the position *x* of the process X(t) corresponds to the number of bytes currently in the buffer. The number of bytes received at a unit of time is a product of two independent random variables: *X*—the number of packets; and *Y*—the size of packets. The mean of a product variable XY is E(XY)=E(X)E(Y) and the variance is

$$\begin{array}{ccc} Var(XY) & = & E\left(X^{2}Y^{2}\right)-{\left(E\left(XY\right)\right)}^{2}\\ & = & Var\left(X\right)Var\left(Y\right)+Var\left(X\right){\left(E\left(Y\right)\right)}^{2}\\ & + & Var\left(Y\right){\left(E\left(X\right)\right)}^{2}, \end{array}$$

the mean number of arrived at a time unit packets is E(X)=λ and the variance is $Var\left(X\right)=\lambda^{3}\sigma_{A}^{3}$, and we denote by *m* the mean size of a packet (in bytes) and by $\sigma_{m}^{2}$ the variance of its size, therefore the mean number of arrived at a time unit bytes is

β=λm

and the variance of number of arrived at a time unit bytes defining α in Equation (1) is

(2) $$\alpha =\lambda^{3}\sigma_{A}^{2}\sigma_{m}^{2}+\lambda^{3}\sigma_{A}^{2}m^{2}+\sigma_{m}^{2}\lambda^{2}.$$

We consider the unlimited queue; therefore, the diffusion process is limited only by a reflecting barrier at x=0 (the queue is never negative).

Without any barrier, the density of the unrestricted process defined by Equation (3) and started at $x_{0}$ is

(3) $$f\left(x,t;x_{0}\right)=\frac{1}{\sqrt{2\pi \alpha t}}\exp\left[-\frac{{\left(x-x_{0}-\beta t\right)}^{2}}{2\alpha t}\right].$$

### 4.2. Modelling of the Time-Based Packet Aggregation Process

When the value of waiting time of the first packet in the buffer reaches a defined maximum waiting time threshold, then the content of the buffer is aggregated into a larger packet and the time is reset to zero. The growth process of the number of bytes accumulated after time *t*, can be approximated by a diffusion process that starts at x=0 at time t=0 and with an absorbing barrier at x=N (the maximum defined size threshold, and for a time-based packet aggregation mechanism, this value is set large enough that the time threshold is always reached). A diffusion process that starts at x=0 and is absorbed at x=N has pdf ϕ(x,t) [50]

(4) $$\begin{array}{c} \phi \left(x,t\right)=\frac{1}{\sqrt{2\pi \alpha t}}\{\exp\left[-\frac{{\left(x-\beta t\right)}^{2}}{2\alpha t}\right]-\\ \exp\left[\frac{2\beta N}{\alpha }-\frac{{\left(x-2N-\beta t\right)}^{2}}{2\alpha t}\right]\}. \end{array}$$

At the moment of reaching the defined maximum waiting time threshold *T*, the probability density function of the diffusion process after is ϕ(x,T)

When the content of the buffer is aggregated into a larger packet, the timer is reset to t=0 and is activated again when a new small packet arrives into the packet buffer, and the accumulation process starts again till the waiting time threshold is reached. Suppose that the first packet that arrives into the buffer to trigger the accumulation process is of random size of *M* bytes, with pdf $f_{M}\left(x\right)$, then the actual number of the aggregated packet (the larger packet) is $X_{B}=X+M$, and its pdf is

(5) $$f_{X_{B}}=\phi \left(x,T\right)*f_{M}\left(x\right);$$

its mean and variance are, respectively, $\mu_{X_{B}}=\mu_{X}+\mu_{m}$ and $\sigma_{X_{B}}^{2}=\sigma_{X}^{2}+\sigma_{M}^{2}$.

**Figure 9:**
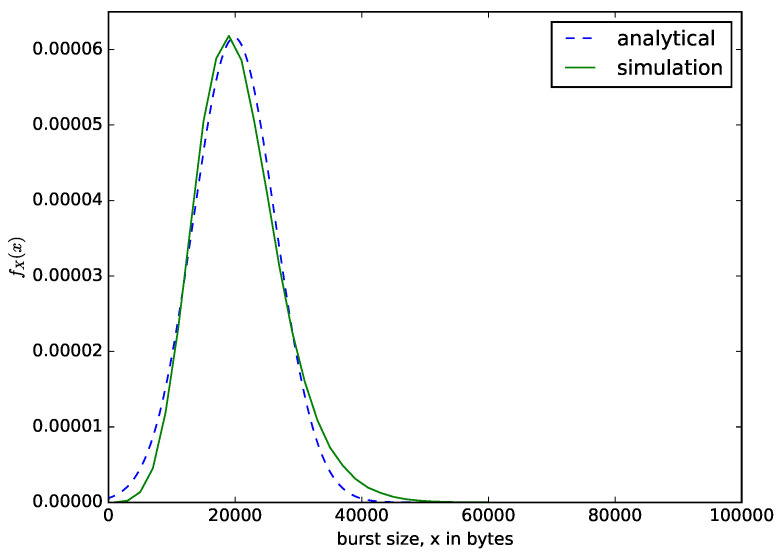
The distribution of the size of the aggregated packet in bytes for a time-based packet aggregation mechanism.

Figure 9 shows the distribution of the sizes of the aggregated packets in bytes. We set the size threshold or buffer size as *N* = 100,000 bytes to ensure that only the maximum waiting time threshold criteria is satisfied. The value of the maximum waiting time threshold is T=0.02 s. The value of *T* should be carefully chosen to ensure that the size of the aggregated packets do not exceed the maximum transmission unit of the target network, whose throughout efficiency is improved by the packet aggregation. For example, in aggregating voice or IoT packets to be transmitted over IP network, the value of *T* should ensure that the sizes of the aggregated packets should not exceed 1500 bytes. We compare the result from the analytical model of Equation (4) with the results from the simulation. Since the small packets accumulated in the buffer are aggregated into a larger packet when the waiting time threshold is reached, regardless of the number of bytes present in the buffer, the sizes of the aggregated packets dispatched varies. The sizes of the aggregated packets depend on the rate at which the packets are arriving into the buffer, the sizes of the arriving packets and the value of the waiting time threshold, *T*. If the sizes of the aggregated packets are small, we have low throughput, and when they are too large, we have high throughput, but very large sizes of aggregated packets could lead to packet losses in the core network.

Since the content of the buffer is aggregated into a larger packet only when the waiting time threshold is reached, then the time from when the current threshold is reached, and the buffer content is aggregated to the moment when the next one occurs (the interdeparture time) is $t_{a}+T$ where $t_{a}$ is the interarrival time. The accumulation time *T* is constant, but the interdeparture time is random because it depends on the interarrival time, since the timer is triggered only when the first packet arrives. The pdf of the interdeparture times is $f_{D}\left(t\right)=f_{A}\left(t_{a}-T\right)$, where $f_{A}\left(t_{a}\right)$ is the pdf of the interarrival times. Suppose that the interarrival time is exponentially distributed then, the interdepature times are exponentially distributed but shifted by T as in [24], e.g.,

(6) $$f_{D}\left(t\right)=\lambda e^{\lambda (t_{a}-T)}$$

**Figure 10:**
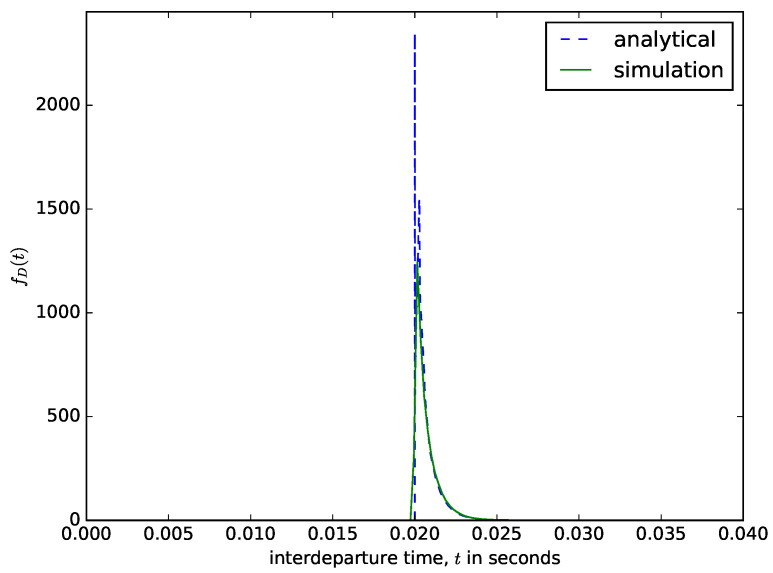
The distribution of the interdeparture times for a time-based packet aggregation mechanism.

Figure 10 shows the distribution of the interdeparture times for a time-based packet aggregation mechanism. The analytical results are obtained by shifting the distribution of the interarrival times from CAIDA by *T*. We compare the interdeparture times from the analytical model with those measured from the simulation in Figure 10. Since the content of the buffer is aggregated when the waiting time threshold is reached, the delay experienced by the packet is fixed, and its value is equal to the value of the waiting time threshold *T*. Since when the buffer is emptied, the next accumulation process starts when the first packet arrives into the buffer, the interdepapature times distribution is similar to the distribution of the interarrival times but shifted by *T* as shown in Figure 10 (e.g., T=0.02).

### 4.3. Modelling of the Size-Based Packet Aggregation Process

For a size-based packet aggregation mechanism, the content of the buffer is aggregated into an aggregated (larger) packet when the maximum size threshold *N* is reached. When a small packet arrives, its size is compared to the difference between the maximum size threshold and the number of bytes accumulated. It is to ensure that after adding the arrived packet to the buffer, the number of bytes accumulated should not exceed the defined maximum size threshold. Therefore, the sizes of the aggregated packets are almost constant as the content of the buffer is aggregated into a larger packet when the size threshold is reached.

The times after which the maximum size threshold is reached varies since the interarrival times of packets into the buffer and the sizes of packets are random. The time from when the first packet arrives in the buffer to when the maximum size threshold is reached and the content of the buffer is aggregated into a larger packet can be considered as the first passage time of the diffusion process from x=0 to x=N. The first passage time of the diffusion process from x=0 to $x=x_{0}$ has pdf [50]

(7) $$\begin{array}{ccc} \gamma_{0,x_{0}}\left(t\right) & = & \lim_{x\to x_{0}}\;\left[\frac{\alpha }{2}\frac{\partial f(x,t;x_{0})}{\partial x}-\beta f\left(x,t;x_{0}\right)\right]\\ & = & \frac{x_{0}}{\sqrt{2\pi \alpha t^{3}}}\exp\left[-\frac{{\left(x_{0}-\beta t\right)}^{2}}{2\alpha t}\right]\;. \end{array}$$

Therefore interdeparture time, which is the first passage time from x=0 to x=N, has pdf $f_{D}\left(t\right)$

(8) $$\begin{array}{ccc} f_{D}\left(t\right) & = & \frac{N}{\sqrt{2\pi \alpha t^{3}}}\exp\left[-\frac{{\left(N-\beta t\right)}^{2}}{2\alpha t}\right]\;. \end{array}$$

**Figure 11:**
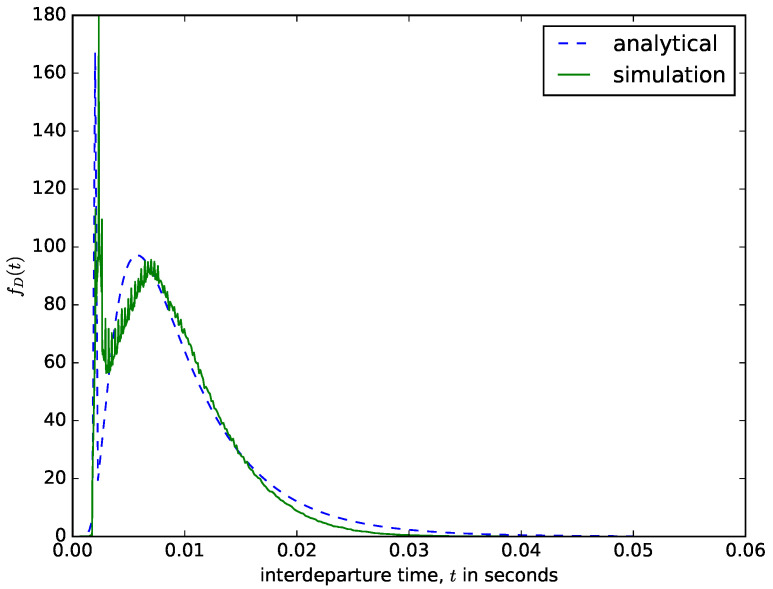
The distribution of the interdeparture times for a size-based packet aggregation mechanism.

Figure 11 shows a comparison of the interdeparture times from the diffusion approximation model and the ones obtained using a discrete-event simulator. The value of the threshold size should be carefully chosen to ensure that the delay experienced by the first packet that arrived into the buffer should not be too high. When the packets are aggregated, overhead bytes are added to them before sending the entire packet to the transmission module. Therefore, when choosing the value of *N*, the designer should bear in mind the value of the maximum transmission unit for the network over which the aggregated packet needs to be transmitted. The results presented in Figure 11 were obtained for *N* = 10,000 bytes. When aggregating the voice traffic from mobile networks or smaller packets from IoT and wireless sensor networks, the value of *N* can be 1497 bytes, since the Ethernet maximum transmission unit is 1500 bytes (the rest is used for overhead bytes) [16]. Since the content of the buffer is emptied when the maximum size threshold is reached, regardless of the waiting time of the packets in the buffer, the waiting times of the packets in the buffer vary and are distributed as shown in Figure 11. The waiting time experienced by the first packet that arrives into the buffer depends on the interarrival times of packets into the buffer, the packet sizes, and the value of the maximum size threshold *N*. If larger packets arrive at a faster rate (very short interarrival times), then the waiting time threshold will be reached very fast, accounting for the sharp spike in Figure 11.

### 4.4. Modelling of Hybride Packet Aggregation Process

For a hybrid packet aggregation mechanism, the content of the buffer is aggregated into a larger packet when either the number of bytes in the buffer is greater than or equal to the maximum size threshold or when the waiting time of the first packet in the buffer reaches the maximum waiting time threshold. Therefore, the values of *N* and *T* can be selected in such a way as to ensure any of the aggregation criteria is reached. Figure 8 shows a comparism of the analytical and the simulation results of the interdeparture times for a hybrid packet aggregation mechanism.

**Figure 12:**
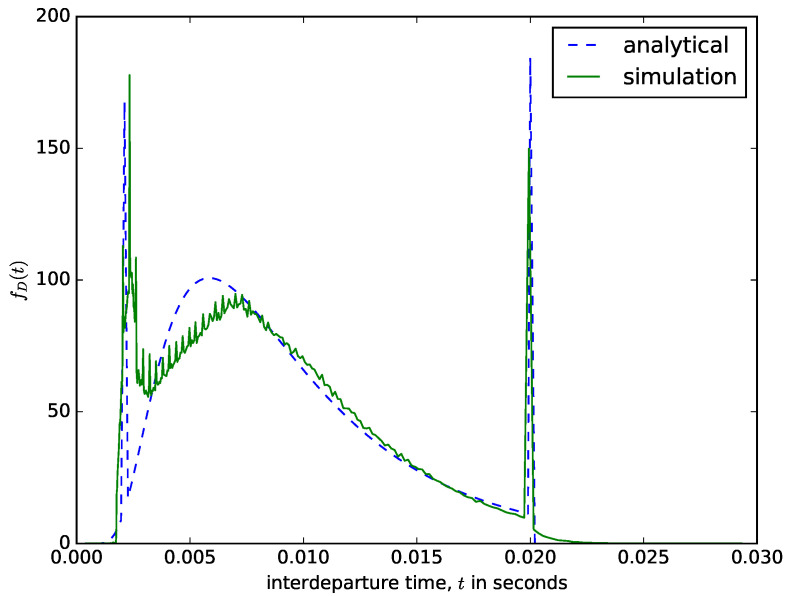
The distribution of the interdeparture times for a hybrid packet aggregation mechanism.

The sharp spike at the start of the interdeparture time distribution in Figure 12 is due to the frequent attainment of the maximum size threshold (perhaps due to fast arrivals or arrivals of packets with larger sizes). These spikes are very visible in the distribution from the simulation but analytically is approximated as the probability that the first passage time is less than or equal to the minimum filling time $t_{min}$, e.g.,

$$F_{D}\left(t\leq t_{min}\right)=\int_{0}^{t_{min}}f_{D}\left(t\right)dt,$$

where $t<\frac{N-M_{max}}{\beta }$, and $M_{max}$ is the maximum size of the arrival packets. The sharp spike at the end of the distribution is due to the frequent attainment of the maximum time threshold. This is the probability density that the diffusion process will end exactly when the deadline *T* is reached and is given by:

(9) $$\begin{array}{c} \int_{T}^{\infty }\gamma_{0,N}\left(t\right)dt=N\{2-\mathrm{erfc}\left(\frac{N-T\beta }{\sqrt{2T\alpha }}\right)+\\ e^{\frac{2N\beta }{\alpha }}\mathrm{erfc}\left(\frac{N-T\beta }{\sqrt{2T\alpha }}\right)\} \end{array}$$

where

$$\mathrm{erfc}\left(t\right)=1-erf\left(t\right),\;\mathrm{erf}\left(t\right)=\frac{2}{\sqrt{\Pi }}\int_{0}^{t}e^{-\xi^{2}}d\xi .$$

In the case of the hybrid algorithm, the design parameters are the maximum burst size and the maximum time or deadline. Some design criteria such as the probability that the maximum burst size threshold is reached and the probability that the maximum time threshold is reached could be used to choose the design parameters, *N* and *T*. The probability of that time threshold will be reached, and the probability that the maximum burst size threshold will be reached, respectively, are

(10) $$F_{D}\left(t\leq T\right)=\int_{0}^{T}f_{D}\left(t\right)dt\;F_{X_{B}}\left(x_{B}\leq N\right)=\int_{0}^{N}f_{X_{B}}\left(x_{B}\right)dx_{B}$$

If the value of the parameters *N* and *T* are chosen such that the probability that the time threshold is reached is 0.99, then the assembler is an MT assembler and if they are chosen such that the probability that the maximum burst size is reached is 0.99, then the assembler is an MS assembler [24]. The proposed diffusion approximation based performance analysis models for the time-based, size-based, and hybrid packet aggregation mechanism do not make any assumption about the distribution of the interarrival times of packets into the buffer and the distribution of the packet sizes as in most of the existing studies. Therefore, the distribution of the interarrival times and that of packet sizes is general; that is, any distribution can be used, including the distribution from traffic measurements such as those used in this studies.

## 5. Performance Analysis of a Slot-Based Packet Aggregation Mechanisms

In a slot-based packet aggregation mechanism, at the end of each time slot Δ we decide on the aggregation of packets, following the ruls explained below. It is a suitable packet aggregation mechanism in a wireless network environment in which access to the channel is shared by multiple devices, and each device is assigned a defined timeslot for transmission. A slot-based packet aggregation mechanism for enhancing VoIP performance on IEEE 802.11 wireless mesh networks was discussed in [52]. A novel slot-based packet aggregation scheme for the aggregation IP packets in Next Generation of Routers for Energy Efficiency Networks (N-GREEN) optical metro networks was proposed in [20,21]. In this section, we present the performance evaluation models for a slot-based packet aggregation scheme in an N-GREEN network, previously considered by us [31].

### 5.1. The Slot-Based Packet Aggregation Mechanism

In the context of the N-GREEN (Next Generation of Routers for Energy Efficiency Networks), smaller electronic packets called Service Data Units (SDUs) from access networks (e.g., DSL, wired and wireless LANs, 2G/3G/4G/5G mobile networks, and IoT networks), are stored following a First-in-First-out (FIFO) queueing discipline. At every time slot allocated to the buffer, the SDUs (mtheir mean size is *m*) are aggregated into larger packets called Packet Data Units (PDUs) of the size *L* that are converted into optical packets and then inserted into the optical transmission system, provided that the optical transmission channel is not occupied.

**Figure 13:**
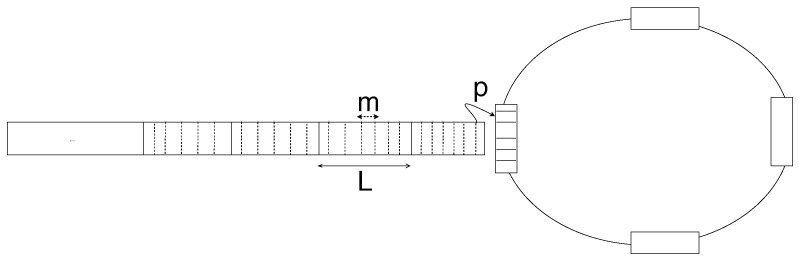
The N-GREEN packet aggregation system.

Figure 13 illustrates the mechanism at N-GREEN in which at every time slot Δ, where PDUs are inserted into any of the empty containers circulating in the ring. If the available container is empty (with a probability *p*), then the PDU is inserted into it. Otherwise, the PDU waits for the next time slot to try again.

The containers are circulating a ring, and each buffer expects the arrival of a container after every Δ seconds, and in a system with multiple buffers, a container can be empty or occupied with PDU loaded by another buffer. Therefore the PDUs are loaded or inserted by the buffers and they are converted into optical packets and then they are unloaded and transmitted by the transmission unit. Consider the following cases of aggregating the SDUs into a PDU and inserting the PDU into the containers at each time slot:

1 First case: At every time slot, the SDUs are aggregated and inserted into an empty container. If the number of bytes stored in the buffer is less than L bytes, all of its content is aggregated into a PDU and inserted into the container. However, if the number of bytes stored in the buffer is larger than *L* bytes, then the SDUs are aggregated into a PDU of size *L* bytes and inserted into the container, and the rest of the SDUs in the buffer waits for the next available container in the next time slot.
2 Second case: At every time slot, the SDUs are aggregated into a PDU and inserted into a container only when the number of bytes stored in the buffer is greater than or equal to *L* bytes.

We modeled the packet aggregation mechanism for a single buffer and assumed that the behaviour is the same for all the buffers. We present a diffusion approximation based performance evaluation model for a slot-based packet aggregation mechanism.

### 5.2. Diffusion Approximation Model for a Slot-Based Packet Aggregation Mechanism

As the SDUs arrive into the buffer, the number of bytes grow continuously. We represent the dynamic changes in the number of bytes stored in the buffer by a diffusion process. At each time slot, when the SDUs are aggregated into a PDU and inserted into a container, and the number of bytes in the buffer reduces instantaneously by an amount equivalent to the size of the SDU. We approximate the growth of the number of bytes in the buffer by a reflecting barrier at x=0 (as the number of bytes stored in the buffer must be positive), its pdf is [50]

(11) $$f\left(x,t;x_{0}\right)=\frac{1}{\sqrt{2\pi \alpha t}}\left[a(t)-\exp(2\beta x/\alpha )b(t)\right].$$

where

$$a\left(t\right)=\exp\left[-\frac{{\left(x-x_{0}-\beta t\right)}^{2}}{2\alpha t}\right],\;b\left(t\right)=\exp\left[-\frac{{\left(-x-x_{0}-\beta t\right)}^{2}}{2\alpha t}\right]$$

After the PDU is inserted into the container at each time slot, the diffusion process jumps back and starts at a new initial point. We may define the initial condition in a more general way, the starting point is not only at $x_{0}$, but it is at any point ξ given by a distribution ψ(ξ), in this case

(12) $$f\left(x,t;\psi \right)=\int_{0}^{\infty }f\left(x,t;\xi \right)\psi \left(\xi \right)d\xi .$$

When the PDU is inserted into the container, the decrease in the number of bytes in the buffer corresponds to an instantaneous jump back of the diffusion process X(t). Therefore we concentrate on the diffusion description during constant intervals Δ and the definition of immediate changes of the process between these intervals. Below, we consider the two cases of aggregating SDUs into a PDU which is inserted into the container.

### 5.3. Case 1

At each time slot, if the number of bytes *x* present in the buffer is less than or equal *L* bytes (that is x≤L), then all of the content of the buffer is aggregated into a PDU and inserted into the container with a probability *p*, otherwise, it is inserted during the next time slot. Therefore, for x≤L, the entire content of the buffer is aggregated and inserted into the container, corresponding to a jump back of the diffusion process to x=0, and continue to increase again as the queue of SDUs grows with the arrival of more SDUs into the buffer. However, if the number of bytes in the buffer is greater than *L* bytes (that is x>L), then the SDUs are aggregated into a PDU of size *L* and inserted into the container, and the number of bytes in the buffer will decrease by *L* bytes, corresponding to a jump back of the diffusion process to x=x−L, and starts to increase again as the number of bytes in the buffer grows with the arrival of new SDUs. We treat the diffusion process from a given starting point till the point when it jumps back after the insertion of a PDU into the container during the next timeslot; that is we study the dynamic changes in the number of bytes in the buffer during the time interval Δ between timeslots. When the diffusion process jumps back to a given point after the insertion of the PDU into the container, that point becomes the starting the diffusion process that approximates the growth of number of bytes in the buffer.

Denote by $f^{\left(i\right)}\left(x,\Delta ;\psi^{\left(i\right)}\right)$ the pdf of the process during *i*th interval Δ. At the beginning of each interval, the time is set to zero, hence always t∈[0,Δ]. The distribution of the number of bytes in the buffer at the end of each Δ, after the jump, if it occurs, defines the initial distribution of the number of bytes stored in the buffer for the next timeslot. Assume that the initial value of the process is $x_{0}=0$, i.e., the buffer is empty. At the end of the first interval, the position of the process, before a possible jump, is given by $f^{\left(1\right)}\left(x,\Delta ;0\right)$. The jump occurs with probability *p* giving the initial distribution for the next interval

(13) $$\psi^{\left(2\right)}\left(0\right)=\int_{m}^{L}f^{\left(1\right)}\left(x,\Delta ;m\right)dx$$

and for ξ>0

(14) $$\psi^{\left(2\right)}\left(\xi \right)=f^{\left(1\right)}\left(\xi +L,\Delta ;0\right)$$

or with probability 1−p there is no jump and the new initial condition is given by the position of the process at the end of previous time-slot

(15) $$\psi^{\left(2\right)}\left(\xi \right)=f^{\left(1\right)}\left(\xi ,\Delta ;0\right).$$

Therefore, the complete initial condition for the second time slot is defined as

(16) $$\begin{array}{ccc} \psi^{\left(2\right)}\left(0\right) & = & p\int_{m}^{L}f^{\left(1\right)}\left(x,\Delta ;m\right)dx,\;\\ \psi^{\left(2\right)}\left(\xi \right) & = & pf^{\left(1\right)}\left(\xi +L,\Delta ;0\right)\\ & + & \left(1-p\right)f^{\left(1\right)}\left(\xi ,\Delta ;0\right),\;\xi >0 \end{array}$$

and these initial conditions determine the movement of the process during the second time slot and its position at the end of it, $f^{\left(2\right)}\left(\xi ,\Delta ;\psi^{\left(2\right)}\right)$.

**Figure 14:**
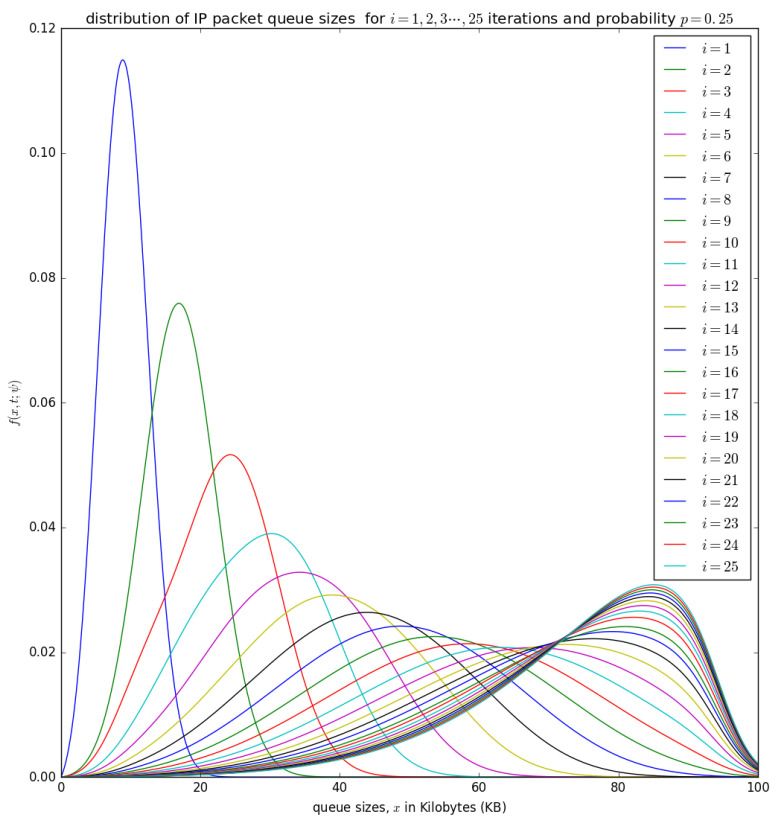
The distribution of the aggregation queue size, f(x,t;ψ) if p=0.25 for consecutive iterations as in Equation (17) i=1…25.

**Figure 15:**
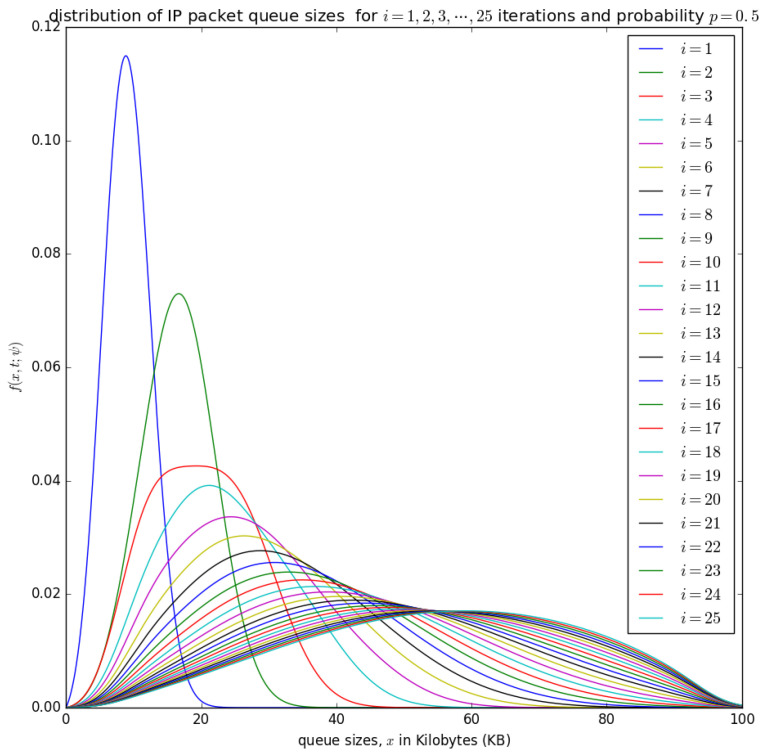
The distribution of the aggregation queue size, f(x,t;ψ) if p=0.5 for consecutive iterations as in Equation (17) i=1…25.

**Figure 16:**
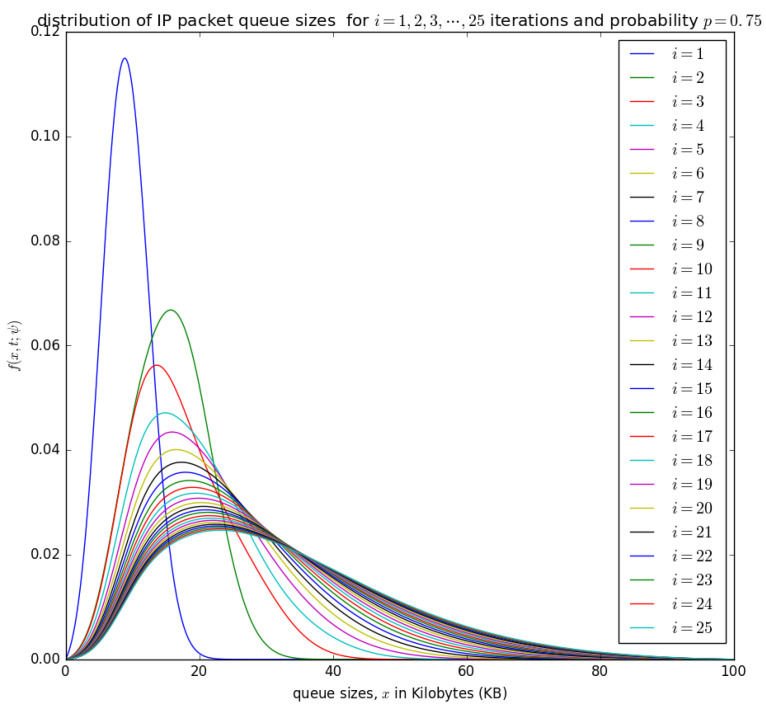
The distribution of the aggregation queue size, f(f(x,t;ψ)) if p=0.75 for consecutive iterations as in Equation (17) i=1…25.

**Figure 17:**
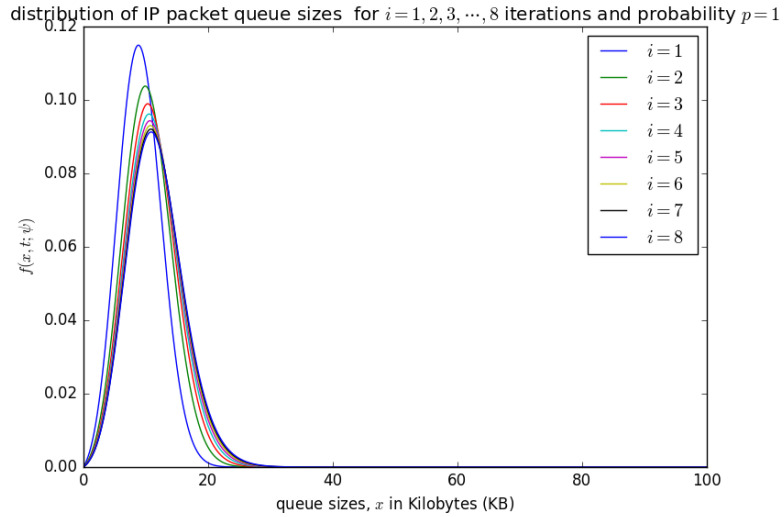
The distribution of the aggregation queue size, f(x,t;ψ) if p=1 for consecutive iterations as in Equation (17) i=1…25.

In the same way for the next slots,

(17) $$\begin{array}{ccc} \psi^{\left(n+1\right)}\left(0\right) & = & p\int_{0}^{L}f^{\left(n\right)}\left(x,\Delta ;\psi^{\left(n\right)}\right)dx,\;\\ \psi^{\left(n+1\right)}\left(\xi \right) & = & pf^{\left(n\right)}\left(\xi +L,\Delta ;\psi^{\left(n\right)}\right)\\ & + & \left(1-p\right)f^{\left(n\right)}\left(\xi ,\Delta ;\psi^{\left(n\right)}\right),\;\xi >0 \end{array}$$

until the convergence, when $\psi^{\left(n+1\right)}\left(\xi \right)=\psi^{\left(n+1\right)}\left(\xi \right)$ and $f^{\left(n+1\right)}\left(x,t;\psi^{\left(n+1\right)}\right)\approx f^{\left(n\right)}\left(x,t;\psi^{\left(n\right)}\right)$. This convergence is illustrated later in Figure 14, Figure 15, Figure 16 and Figure 17 for various valuess of *p*.

Since when the time slot occurs, and the number of bytes stored in the buffer is less than *L* bytes, the content of the buffer should be aggregated and inserted into the container, the sizes of the PDU may be less than *L*. Smaller PDU (aggregated packet) sizes result in lower aggregation throughput efficiency, as the objective is to have more SDUs aggregated into a larger PDU and share the same header bytes during transmission, hence reducing protocol overheads. The probability of inserting a PDU of size *L* is $\int_{L}^{\infty }f\left(x,\Delta ;\psi \right)dx$, but the probability of inserting a PDU of size x<L is f(x,Δ;ψ). Therefore, the mean effective size of the packet is

(18) $$L_{\mathrm{eff}}=L\int_{L}^{\infty }f\left(x,\Delta ;\psi \right)dx+\int_{0}^{L}f\left(x,\Delta ;\psi \right)xdx.$$

The aggregation ratio is

(19) $$\epsilon =\frac{L_{\mathrm{eff}}}{m}$$

where *m* is the mean size of an SDU.

### 5.4. Case 2

At the occurrence of the time slot after the time interval Δ, the SDUs are aggregated into a PDU of size *L* bytes and inserted into the container only is the number of bytes in the buffer is greater than or equal to *L* bytes (that is x≥L). The equations of Case 1 are adapted in the following way. As previously, at the end of the first interval Δ the pdf of the number of bytes stored in the buffer is $f^{\left(1\right)}\left(x,\Delta ;0\right)$, and for any slot n≥1

(20) $$\begin{array}{ccc} \psi^{\left(n+1\right)}\left(\xi \right) & = & f^{\left(n\right)}\left(\xi ,\Delta ;\psi^{\left(n\right)}\right),\;\xi <L,\\ \psi^{\left(n+1\right)}\left(\xi \right) & = & pf^{\left(n\right)}\left(\xi +L,\Delta ;\psi^{\left(n\right)}\right),\\ & + & \left(1-p\right)f^{\left(n\right)}\left(\xi ,\Delta ;\psi^{\left(n\right)}\right),\;\xi \geq L. \end{array}$$

When the steady state is reached, the initial distribution $\psi =\lim_{n\to \infty }\psi^{\left(n\right)}$ and the density of the number of bytes stored in the buffer at the end of Δ is the same, e.g., f(x,Δ;ψ)=ψ(x).

The aggregation ratio is

(21) $$\epsilon =\frac{L}{m}$$

Since the SDUs are aggregated into a PDU only when the number of bytes in the queue is greater than or equal to *L* bytes, the sizes of the PDUs that are converted into optical packets and transmitted are fixed. This ensures that the sizes of the PDU can be chosen by the designer such that it does not exceed the maximum transmission unit and the small PDUs that create throughput inefficiency in the transmission core network is avoided. Therefore, the designer or the network operator has control over the throughput, but in case one the throughput varies slightly as the size of the PDU can be less than *L* (when the time slot arrives and the number of bytes in the buffer is less than *L* bytes, the content of the buffer is aggregated into a PDU).

### 5.5. Queueing Delay

One of the major aims of packet aggregation is to improve throughput efficiency by reducing protocol overhead. The advantage of slot-based packet aggregation mechanism over other packet aggregation mechanism is that it produces higher throughput as the designer has control over the size of the PDU, *L*. That is, when the value of *L* is larger, more SDUs can be aggregated into a PDU and transported with just one header, which ensure that a large payload is transported with a relatively small overhead. However, high throughput is achieved at the cost of longer delays as some packets may wait for too long in the buffer. The major parameters that influence the delay are the time interval between time slots (the time from when a buffer tries to insert the PDU into the container and the next trial), Δ and the channel availability probability (the probability of inserting the PDU into the container at a given time slot), *p*.

When an incoming SDU arrives into the buffer in which other SDUs that arrived earlier have been stored, it joins the queue at the tail end. We assume that all SDU (small packets) are treated the same without any prioritisation: When a SDU arrives it joins the queue at the tail end (SDUs join the queue sequentially). It should be noted that in some real implementation of this mechanism, the SDUs could be queued up based on their time sensitivity such that SDUs belong to real-time applications can be shuffled to the front (head) of the queue, aggregated, and inserted into the queue to ensure that they are transported immediately to satisfy their quality of service (QoS) requirements. However, to keep our analysis tractable, we have assumed that all SDUs have the same priority.

Suppose that an arriving SDU that arrives at time t∈(t,Δ) sees the queue distribution f(x,t;ψ). With the probability

$$p_{1}=\int_{0}^{L}f\left(x,t,\psi \right)dx$$

the number of bytes in the buffer is less than *L*. After the time interval Δ, the SDUs are aggregated into a PDU and inserted into the container with a probability *p* (if the container is empty), otherwise, it waits for the next time slot after the same time interval (e.g., Δ). Therefore, its waiting time will be Δ−t with probability *p* or Δ−t+Δ with probability (1−p)p, or Δ−t+2Δ with probability ${\left(1-p\right)}^{2}p$, …Δ−t+nΔ with probability ${\left(1-p\right)}^{n}p$ depending on the earliest arrival of a time slot with an empty container. This probability follows a geometric distributed, and its distribution density function is denoted as

(22) $$\begin{array}{ccc} f_{W1}\left(w,t\right) & = & p\delta \left(w-(\Delta -t)\right)+\left(1-p\right)p\delta \left(w-\left(2\Delta -t\right)\right)\\ & & +{\left(1-p\right)}^{2}p\delta \left(w-\left(3\Delta -t\right)\right)+\ldots \\ & & +{\left(1-p\right)}^{n}p\delta \left(w-\left(\left(n+1\right)\Delta -t\right)\right)+\ldots \end{array}$$

where δ(x) is Dirac delta function.

Assuming that the SDU arrival may happen at any moment *t* of the time slot with the same density 1/Δ, we determine $f_{W1}\left(w\right)$ as

(23) $$f_{W1}\left(w\right)=\int_{0}^{\Delta }\frac{1}{\Delta }f_{W1}\left(w,t\right)dt.$$

Similarily, if the queue size is between *L* and 2L which will happen with probability

$$p_{2}=\int_{L}^{2L}f\left(x,t;\psi \right)dx$$

then we should have two empty containers to insert two PDU in two consecutive time slots. It means that we add the delay incured by waiting for the arrival of the second empty container in second time slot to the waiting time (for the first time slot) defined above, This delay is equal to Δ with probability *p* if just the next container is empty, 2Δ if the next container is occupied but the one after it is empty—with probability (1−p)p, etc. The distribution of this additional delay $f_{\Delta }\left(w\right)$ is

(24) $$\begin{array}{ccc} f_{\Delta }\left(w\right) & = & p\delta (w-\Delta )+(1-p)p\delta (w-2\Delta )+\\ & & +{\left(1-p\right)}^{2}p\delta \left(w-3\Delta \right)+\ldots \\ & & +{\left(1-p\right)}^{n}p\delta \left(w-\left(n+1\right)\Delta \right)+\ldots \end{array}$$

Therefore, the waiting time for a SDU that arrives at time *t* and seeing the queue size between *L* and 2L is determined by the convolution

$$f_{W2}\left(w\right)=f_{W1}\left(w\right)*f_{\Delta }\left(w\right)$$

and the waiting time for the arriving SDU that sees the queue size between 2L and 3L is determined by

$$f_{W3}\left(w\right)=f_{W1}\left(w\right)*f_{\Delta }\left(w\right)*f_{\Delta }\left(w\right)$$

and the waiting time for an arriving SDU that sees the queue size between (n−1)L and nL (i.e., the SDU is loaded at the *n*th timeslot) is

(25) $$f_{Wn}\left(w\right)=f_{W1}\left(w\right)*f_{\Delta }{\left(w\right)}^{*(n-1)}$$

The probabilities $p_{n}$, n=1,… that an arriving SDU joins the queue and sees the queue size between x∈[(n−1)L,nL] is

(26) $$p_{n}=\int_{(n-1)L}^{nL}f\left(x,t;\psi \right)dx$$

Therefore, an SDU that arrives and joins a queue that is longer will wait longer, and its waiting time also depends on the probability that the circulating container that arrives to its buffer at the *n*th timeslot is empty as shown in Figures 19 and 20 in the next section below.

### 5.6. Numerical Examples

In numerical examples we use PDUs of length L=12.5 KB (12,500 bytes) and the time slots Δ=10 μsec at 10 Gb/sec, the same realistic parameters as considered in [20,21]. The interarrival times have a general distribution with mean 1/λ, variance $\sigma_{A}^{2}$, and the size of electronic packets is determined by a general distribution having density with mean *m* and variance $\sigma_{m}^{2}$. Assume λ=1 packet/μsec, the average packet size m=700 bytes, squared coefficients of variation $C_{A}^{2}=\sigma_{A}^{2}\lambda^{2}=1$ and $C_{m}^{2}=\sigma_{m}^{2}/m^{2}=1$. It means that the parameters of the diffusion equation are: Arrival rate β=λm=0.7 kB/μsec and α=1.47, as defined by Equation (2)

Naturally, the variances $C_{A}^{2}$, $C_{m}^{2}$ may be different and represent any distribution, it is the advantage of diffusion approximation. Note that the squared coefficient of variation close to one does not mean necessarily that a distribution is resembling the exponential one. When analysing the distributions of packet sizes and times between packets given by CAIDA (Center for Applied Internet Data Analysis) repositories, we met distributions that are far away from exponential ones, but with $C^{2}\approx 1$. The results presented are based on the PDU insertion mechanism in case 1.

Figure 14, Figure 15, Figure 16 and Figure 17 illustrate the convergence of the solution formulated in Equation (17) for various values of *p*, it is visible that at each case, 25 iterations give satisfactory results.

Figure 14 shows the distribution of the number of bytes in the aggregation buffer. Initially, the buffer is zero, and after the accumulation time Δ, the distribution of the number of bytes in the buffer is represented by a diffusion process that starts at x=0 and grows as more packets arrive into the buffer (see the blue curve for i=1) in Figure 14. At the occurrence of the first timeslot, small packets in are aggregated into a larger packet of size *L* and inserted into the container with a probability p=0.25, which shifts the diffusion process backwards by x=L to a random point ξ which becomes the new initial point for the diffusion that represent the process that represents the distribution of the number of bytes in the buffer after the shift. At the occurrence of the timeslot, the PDU of size *L* may not be inserted into the container with a probability of 1−p. The distribution of the number of bytes in the buffer after the occurrence of the second timeslot can be represented by a diffusion process that starts at a random point ξ and grows as more packets arrives in the buffer (see the green cure for i=2 in Figure 14). After the 25 timeslots, the distribution obtained after the occurrence of the 25th timeslot is similar to that obtained from the 24th timeslot, which is some form of steady-state convergence behaviour of the distribution of the number of bytes in the buffer.

Figure 15 and Figure 16 show the distribution of the number of bytes in the buffer for various timeslots, and for p=0.5 and p=0.75, respectively. Similar to Figure 14, the distributions for higher timeslots is shifted to the right as the starting point of the diffusion process for higher timeslot may be slightly larger. After 25 timeslots, the distributions converge into a steady-state as in Figure 14. Unlike in Figure 14, the distributions for p=0.5 and p=0.75, respectively, are relatively shifted to the left because the probability of loading the PDU from the buffer to the container is larger.

Figure 17 shows the distribution of the number of bytes in the buffer for p=1. It can be observed that there is no significant shift of the distributions after the occurrence of various timeslots. For p=1, it is certain that at the occurrence of a timeslot, SDUs are aggregated into a PDU and inserted into the container. The queue size for p=1 is not as large as the case for p=0.75, p=0.5, and p=0.25 as the PDU is loaded into the container at the occurrence of every timeslot when p=1. Steady-state convergence is achieved after the 8th timeslot.

**Figure 18:**
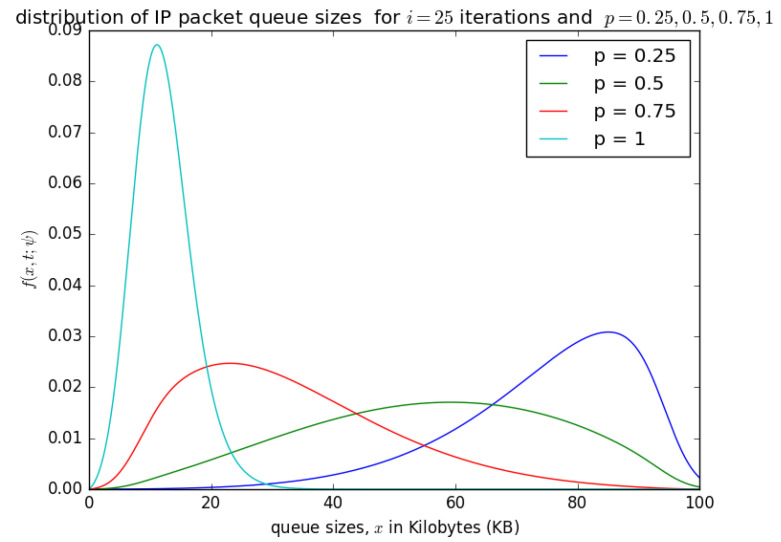
The distribution of the aggregation queue size, f(x,t;ψ) for the i=25 iterations and different values *p*.

Figure 18 presents the impact of probability *p* (probability that at each timeslot, the circulating container that arrives at the buffer is empty) on the final distribution f(x,t;ψ) of the queue length in bytes. If the container is not empty at a given timeslot, we try to load the PDU at the next timeslot. Figure 18 shows that as *p* increases, the queue size in the packet aggregation buffer decreases, because as PDUs are inserted into the container at each timeslot, the queue size decreases. If the probaility *p* (that the circulating containers that arrive at the buffer at each timeslot are empty) decreases, then the PDUs are not inserted into the containers frequently, and the queue size of SDUs in the aggregation increases, and could likely lead to a buffer overflow, even though the aggregation buffer is over-dimensioned.

Table 1 presents probabilities $p_{n}$ that that arriving SDU joins the queue before the interval x∈[(n−1)L,nL] and will be aggregated and inserted into the container after *n* time slots.

**Figure 19:**
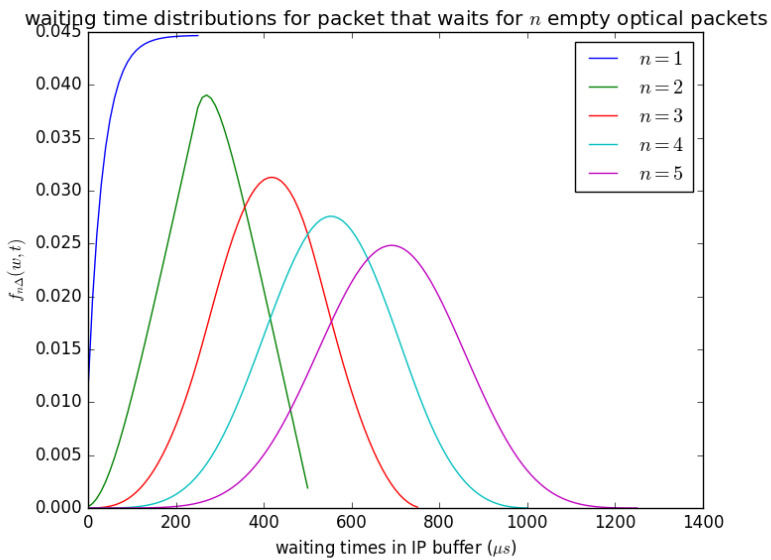
$f_{Wn}\left(w\right)$ as defined in Equation (25); the influence of the number of empty optical packets *n* needed to complete the transfer on the waiting time distribution, p=0.25.

Figure 19 shows the influence of the number of timeslots *n* on the waiting time of an arriving SDU that arrives and sees the queue size between (n−1)L and nL. When an SDU arrives and sees a queue size of about nL, it waits for *n* timeslots, and at each timeslot, SDUs are aggregated to a PDU of size *L* and inserted into the container, provided that the container is empty. The distribution in Figure 19 ($f_{Wn}\left(w\right)$) is obtained from Equation (25). It is described as the waiting time distributions for packets that waits for *n* empty optical packets because the containers that the PDUs are inserted are similar to optical packets in size, and their content is converted to an optical packet and transmitted. The parameter *n* can also be understood as the number of timeslots that will occur for *n* PDUs that contain SDU that arrived earlier to be inserted into available containers before the container that contains the arriving PDU is inserted. Figure 19 shows that as *n* increases, the mean waiting time experienced by arriving SDU increases and can be observed by the distributions’ shifts to the right as *n* increases.

**Figure 20:**
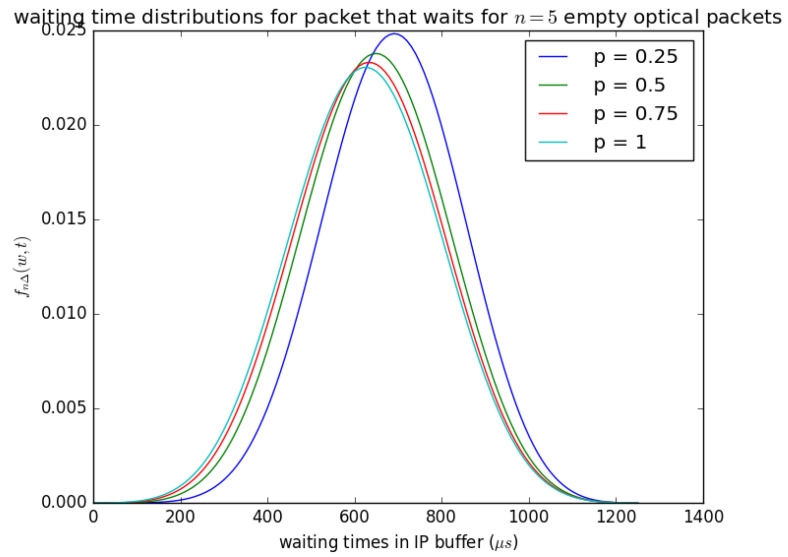
$f_{W5}\left(w\right)$ as defined in Equation (25); the influence of the probability *p* of the empty optical packet on the distribution of waiting time if n=5.

Figure 20 illustrates the influence of the probability *p* that the circulating container that arrives at buffer is empty at each timeslot. If the container that arrives at the buffer in each timeslot and the container that arrives is not empty, the PDU is lot inserted into the container, and it waits for the next timeslot. Figure 20 shows that as the probability *p* that the container is available (i.e., the container is empty) at each timeslot increases, the waiting time experienced by the SDUs decreases, and if *p* is small (the container is not empty) then the waiting time experienced by the SDUs increases. The distribution in Figure 20 ($f_{Wn}\left(w\right)$) is obtained from Equation (25) for n=5 and *p* is varied from 0.25 to 1.

## 6. The Tradeoff between Throughput, Energy Consumption, and Delay

The main goal of sustainable network design is to achieve high throughput and minimise energy consumption with an acceptable QoS (delay, packet losses, and jitter). In this section, we discuss the network performance and energy consumption metrics and how they influence one another in the context of packet aggregation.

### 6.1. The Throughput Efficiency at the Core Network

Generally, the structure of a packet contains three main parts such as the header, the payload, and the trailer. The header contains information required to process a packet (e.g., packet length, synchronisation, protocol, packet identification number, source address, destination address information), the payload contains the actual data delivered from a source user to a destination user, and the trailer which contains information which enables the receiving device to identify the end of the packet and to perform error checking. Since the header and the trailer part of the packet only carries information required to process the packet and not the user data intended to be delivered between two communicating devices, they constitute an overhead to the network and hence, termed overhead bytes. Transporting packets in which a significant proportion (percentage) of the total packet is occupied by overhead bytes results in bandwidth wastage. Suppose that the size of the overhead byte is $O_{b}$ and the average size of the payload is $L_{p}$, then the percentage of the bandwidth wasted due to overhead per packet is

(27) $$\varepsilon_{o}=\frac{O_{b}}{O_{b}+L_{p}}100\%$$

Consider a packet with a header (Ethernet, IPv4, and UDP headers) of 42 bytes, if its payload is 8 bytes (e.g., like the case of IoT packet), then the percentage of the bandwidth consumed by the header (percentage of bandwidth wasted) is 84%. If 100 of such packets are aggregated to share the same header, then the payload size of the aggregated packet is 800 bytes, and the percentage of the bandwidth consumed by the header becomes 5%. It shows that aggregation significantly reduces the percentage of the bandwidth consumed by the headers or overhead bytes. The bandwidth efficiency per packet is

(28) $$\varepsilon_{b}=\frac{L_{p}}{O_{b}+L_{p}}100\%$$

Therefore, aggregating the smaller packets at the edge of the network significantly improves the throughput in the core networks. The more the number of bytes of smaller packets aggregated into larger packets, the higher the bandwidth efficiency.

### 6.2. The Core Network Energy Efficiency

One of the essential benefits of packet aggregation at the edge node is reducing energy consumption in the core network. The energy consumption of the core routers depends on both the number of packets received, processed, and transmitted and on the packet sizes. Packet aggregation reduces the number of packets handled by the core routers, but it increases the packet sizes making the energy benefits offered by packet aggregation not intuitive. It has been shown theoretically and practically in [53,54,55] that the power consumption of a core router or switch consists of a fixed baseline power $P_{B}$ and a dynamic power $P_{D}$. The baseline power is the power consumed by some components such as the cooling Fans, routing engine cards (e.g., during signalling and updating of the routing tables) and other electronic components when they are idle. The dynamic power is the power consumed by the data plane (the line cards and the switching fabric) when it is receiving, processing, and transmitting data packets. Therefore, the power profile of a network router or switch is [53,54,55,56]

(29) $$P=P_{B}+P_{D}$$

**Figure 21:**
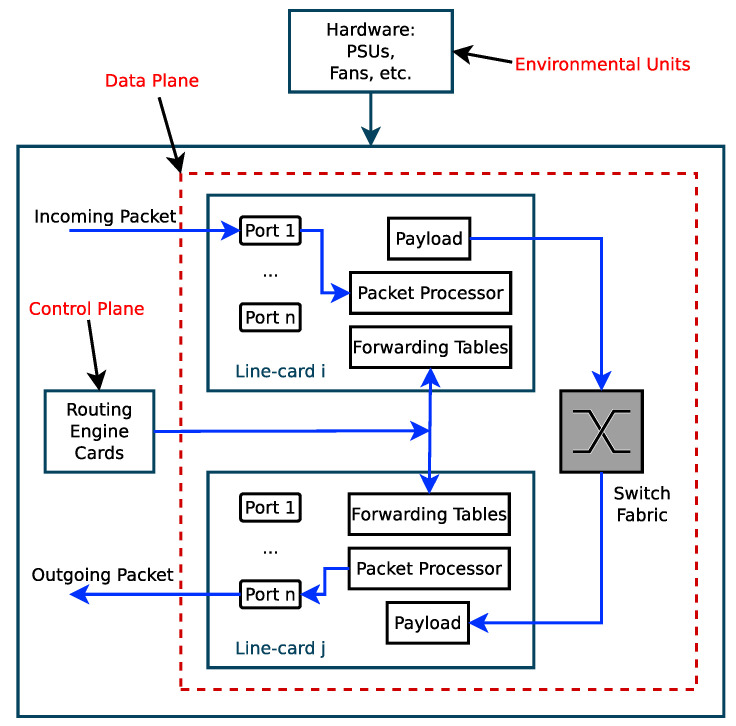
A structure of a Core Router or Switch [53].

Figure 21 shows a simplified router or switch structure considered for theoretical analysis of the power consumption budget of a router or switch. The baseline power is fixed but the dynamic power varies with the number of packets processed per second and on the sizes of packets. The power consumption budget for the *n*th port of a high speed router or switch is

(30) $$P_{n}=PE_{n}+R_{i,n}\left(E_{rx,n}+E_{rs,n}\right)+\lambda_{p,n}E_{p}+R_{o,n}\left(E_{ts,n}+E_{tx,n}\right)$$

where $PE_{n}$ is the power consumed by the *n*th ethernet port when iddle (when there is no traffic on it), $R_{i,n}$ is the number of bytes received per seconds at the *n*th port, $E_{rx,n}$ is the energy required to receive a byte on the ingress Ethernet interface of the *n*th port, $E_{rs,n}$ is the energy required to process and store a byte on the ingress Ethernet interface of the *n*th port, $\lambda_{p,n}$ is the number of packets from the *n*th port processed per second, $E_{p,n}$ is the energy required to process each packet (parsing, route lookup, and forwarding), and it is the same for all packets irrespective of their sizes, $R_{o,n}$ is the number of bytes transmitted per second through the egress Ethernet interface of the *n*th port, $E_{ts,n}$ is the energy required to process and store a byte on the egress Ethernet interface of the *n*th port, and $E_{tx,n}$ is the energy required to transmit a byte on the ingress Ethernet interface of the *n*th port. If the mean packet sizes at the ingress and egress interfaces of the *n*th port are are $m_{i,n}$ and $m_{o,n}$ (in bytes), respectively, then the number of packets received per second through the ingress Ethernet interface of the *n*th port is $\lambda_{i,n}=\frac{R_{i,n}}{m_{i,n}}$ and the number of packets transmitted per second through the egress Ethernet interface of the *n*th port is $\lambda_{o,n}=\frac{R_{o,n}}{m_{o,n}}$ and Equation (30) becomes

(31) $$P_{n}=PE_{n}+m_{i,n}\lambda_{i,n}\left(E_{rx,n}+E_{rs,n}\right)+\lambda_{p,n}E_{p}+m_{o,n}\lambda_{o,n}\left(E_{ts,n}+E_{tx,n}\right)$$

Taking the partial dericatives of Equation (31) with respect to the number of packets received, processed, and transmitted per second, we obtain the energy per packet. The energy per packet for the ingress Ethernet interface of the *n*th port is

$$\frac{\partial P_{n}}{\partial \lambda_{i,n}}=m_{i,n}\left(E_{rx,n}+E_{rs,n}\right)$$

the energy required to process a packet is

$$\frac{\partial P_{n}}{\partial \lambda_{p,n}}=E_{p}$$

It is independent of the packet size as the energy required to process (parsing, route lookup, and forwarding) a small packet and a large packet are the same. The energy per packet for the egress Ethernet interface of the *n*th port is

$$\frac{\partial P_{n}}{\partial \lambda_{o,n}}=m_{o,n}\left(E_{ts,n}+E_{tx,n}\right)$$

The energy per packet for the *n*th port is

(32) $$E_{n,p}=m_{i,n}\left(E_{rx,n}+E_{rs,n}\right)+E_{p}+m_{o,n}\left(E_{ts,n}+E_{tx,n}\right)$$

For $m_{i,n}=m_{o,n}=m_{n}$, then the energy consumption per packet is

(33) $$E_{n,p}=m_{n}E_{rst}+E_{p}$$

where $E_{rst}=E_{rx,n}+E_{rs,n}+E_{ts,n}+E_{tx,n}$. Equation (33) shows that energy per varies linearly with the packet size as demonstrated in [53,54,56] using measurements. Therefore, aggregating smaller packets (e.g., each of size *m*) into larger packets (e.g., each of size $L=\sum_{i=1}^{K}m_{i}$) increases the energy consumption per packet in the core network. It means that by increasing the throughput (increasing the length of the aggregated packet, *L*), the energy consumption per packet at the core network also increases. For a core router or switch with $K_{i}$ idle ports and $K_{a}$ active ports the power consumption budget is

(34) $$P=P_{B}+\sum_{n=1}^{K_{i}}PE_{n}+\sum_{n=1}^{K_{a}}\left[PE_{n}+m_{i,n}\lambda_{i,n}\left(E_{rx,n}+E_{rs,n}\right)+\lambda_{p,n}E_{p}+m_{o,n}\lambda_{o,n}\left(E_{ts,n}+E_{tx,n}\right)\right]$$

The power consumption at the core router and switches increases with an increased number of packets received, processed and transmitted as in Equation (34) which was demonstrated in [53,54,56] using measurements. In order to increase the routing or switching speeds, some core routers and switches contain Ternary Content Addressable Memories (TCAMs) in their hardware which are power-hungry electronic modules, and hence higher energy is required to process a single packet. If the core switches are SDN-based switches, then the power consumption budget considers the power consumed in searching the flow tables, installing the flow rules by the controller, and the communication between the switch and the controller [55]. Therefore, by aggregating a sufficiently large number of small packets into larger packets, the power consumption of the core routers and switches can be significantly reduced compared to increased energy consumption due to an increase in packet size. The core network energy efficiency is [57]

(35) $$\mathrm{Network}\mathrm{energy}\mathrm{efficiency}=\frac{\mathrm{total}\mathrm{useful}\mathrm{traffic}\mathrm{delivered}}{\mathrm{total}\mathrm{energy}\mathrm{consumed}}$$

The objective is deliver more useful traffic (high throughput) with minimum amount of energy possible. Therefore, packet aggregation increases the throughput and reduces the energy consumption in the core network.

### 6.3. Network Delay

When a packet travels through a router or switch, it experiences a delay due to the time required to receive a packet, and the time spent waiting in the input buffer, the required to process the packet (parsing, route lookup, and forwarding), the time spent waiting in the output buffers, and the time required to transmit the packet. The delay budget is

(36) $$D=t_{rx}+t_{ib}+t_{p}+t_{ob}+t_{tx}$$

where $t_{rx}$ is the time required to receive a packet, $t_{ib}$ is the waiting time of the packet in the input buffer if it arrives and joins a queue, $t_{p}$ is the time required to process a packet, $t_{ob}$ is the waiting time of the packet in the output buffers, and $t_{tx}$ is the time required to transmit a packet. For high-speed core routers, $t_{rx}$ and $t_{tx}$ are very small and could be ignored. A detailed analysis of the delay of a network of routers and switches is beyond the scope of this work but have been presented in [29,58].

If a port of an edge router is configured to support packet aggregation, then when the first small packet that needs to be aggregated arrives, it has to wait in the input buffer to be aggregated with other smaller packets into a larger packet. The delay introduced by the aggregation process is significantly larger than the delay experienced by a packet that joins a regular queue and is processed following a defined service discipline (e.g., first-come-first-serve or a priority-based service discipline). Therefore, Equation (36) becomes

(37) $$D=t_{ag}+t_{ib}+t_{p}+t_{ob}+t_{tx}$$

where $t_{ag}$ is the aggregation delay discussed in Section 4 and Section 5 above. The aggregation delay depends on the parameters of the aggregation mechanism deployed, which also influence the throughput and energy consumption. Therefore, a reasonable tradeoff between the throughput, energy consumption, and delay should be made. A recent proposal to attain a reasonable tradeoff between QoS (high throughput and minimum delay) and energy consumption has been presented in [59]. The authors are proposing an SDN approach in which the QoS and energy consumption metrics are estimated and sent to a centralised controller that determines forwarding paths that minimise a goal function consisting of QoS and energy consumption metrics.

## 7. Conclusions

Packet aggregation is a valuable strategy to increase throughput, improve resource utilisation, and reduce energy consumption in access networks, high-speed Internet core networks, and cloud computing data centre networks. The recent increase in the amounts of small packets generated by IoT networks, wireless sensor networks, and 4G/5G mobile networks has increased the need for more research on how to efficiently implement packet aggregation to meet the specific needs of these networks. However, the major drawback of packet aggregation mechanisms is the significant amount of delay that it introduces, making it unsuitable for packets that belong to real-time applications. We have presented a detailed review of packet aggregation applications in access networks (IoT and 4G/5G mobile networks), optical core networks, and cloud computing data centre networks. In the part containing original results, we proposed analytical models for evaluating the performance of packet aggregation mechanisms. They are based on diffusion approximation, where the diffusion process represents the increasing with time content of the aggregation buffer, periodically emptied following aggregation strategy. We have demonstrated the use of measured traffic from real networks to evaluate the performance of packet aggregation mechanisms analytically. We note that it is important to carefully tune the design parameters of the packet aggregation mechanism to obtain a reasonable tradeoff between throughput and energy consumption in the core routers or switches and delay introduced at the edge router or switch by the packet aggregation process.

## Figures and Tables

**Table 1:** Probabilities $p_{n}$, n=1,…,5 that arriving SDU joins the queue before the interval x∈[(n−1)L,nL], as in Equation (26).

$p_{n}$	p=0.25	p=0.5	p=0.75	p=1
n=1	0.000422	0.011993	0.085542	0.5581957
n=2	0.004069	0.066236	0.287785	0.430287
n=3	0.016506	0.130243	0.276826	0.011427
n=4	0.049166	0.183986	0.185722	$8.86\times 10^{-5}$
n=5	0.119076	0.210541	0.100210	$3.66\times 10^{-7}$

## Data Availability

The data is contained in the text of the article.

## References

[1] Cheng Y., Huang M. Based on the Internet of things environment data aggregation security requirements and key technology research. Proceedings of the 2019 International Conference on Information Technology and Computer Application (ITCA).

[2] Deng J., Mark D. An adaptive packet aggregation algorithm for wireless networks. Proceedings of the 2013 International Conference on Wireless Communications and Signal Processing.

[3] Wang S.Y., Wu C.M., Lin Y.B., Huang C.C. (2019). High-speed data-plane packet aggregation and disaggregation by P4 switches. J. Netw. Comput. Appl..

[4] Bonneau V., Copigneaux B. (2017). Industry 4.0 in Agriculture: Focus on IoT Aspects, Digital Transformation Monitor, EU. https://docplayer.net/87939451-Industry-4-0-in-agriculture-focus-on-iot-aspects.html.

[5] Carsten B., Castellani A.P., Zach S. (2012). Coap: An application protocol for billions of tiny internet nodes. IEEE Internet Comput..

[6] Bauer J., Aschenbruck N. Measuring and Adapting MQTT in Cellular Networks for Collaborative Smart Farming. Proceedings of the 2017 IEEE 42nd Conference on Local Computer Networks (LCN).

[7] Vinoski S. (2006). Advanced Message Queuing Protocol. IEEE Internet Comput..

[8] Rao S., Chendanda D., Deshpande C., Lakkundi V. Implementing LWM2M in constrained IoT devices. Proceedings of the 2015 IEEE Conference on Wireless Sensors (ICWiSe).

[9] Khan A., Marwat S.N.K., Ahmed S., Mehmood Y. Packet Aggregation in Mobile Networks for IoT Traffic. Proceedings of the 2019 IEEE 6th International Conference on Engineering Technologies and Applied Sciences (ICETAS).

[10] Marwat S.N.K., Mehmood Y., Khan A., Ahmed S., Hafeez A., Kamal T., Khan A. (2018). Method for Handling Massive IoT Traffic in 5G Networks. Sensors.

[11] Pérez G.O., Hernández J.A., López D.L. Delay analysis of fronthaul traffic in 5G transport networks. In Proceeding of the 2017 IEEE 17th International Conference on Ubiquitous Wireless Broadband (ICUWB).

[12] Tonini F., Khorsandi B.M., Bjornstad S., Veisllari R., Raffaelli C. (2018). C-RAN Traffic Aggregation on Latency-Controlled Ethernet Links. Appl. Sci..

[13] Gowda A., Hernández J.A., López D.L., Kazovsky L. (2017). Delay analysis of mixed fronthaul and backhaul traffic under strict priority queueing discipline in a 5G packet transport network. Trans. Emerg. Tel. Tech..

[14] Saleh A.A.M., Simmons J.M. (2012). All-Optical Networking-Evolution, Benefits, Challenges, and Future Vision. Proc. IEEE.

[15] Bosshart P., Daly D., Gibb G., Izzard M., McKeown N., Ennifer R., Schlesinger C., Talayco D., Vahdat A., Varghese G. (2014). P4: Programming Protocol-Independent Packet Processors. ACM SIGCOMM Comput. Commun. Rev..

[16] Wang S.Y., Li J.Y., Lin Y.B. (2020). Aggregating and disaggregating packets with various sizes of payload in P4 switches at 100 Gbps line rate. J. Netw. Comput. Appl..

[17] Madureira A.L.R., Araújo F.R.C., Sampaio L.N. (2020). On supporting IoT data aggregation through programmable data planes. Comput. Netw..

[18] Lin Y.B., Wang S.Y., Huang C.C., Wu C.M. (2018). The SDN Approach for the Aggregation/Disaggregation of Sensor Data. Sensors.

[19] Choi J., Vu H.L., Cameron C.W., Zukerman M., Kang M., Kahng H.K., Goto S. (2004). The Effect of Burst Assembly on Performance of Optical Burst Switched Networks. Lecture Notes in Computer Science.

[20] Kamli A., Atmaca T., Lepers C., Rataj A., Amar D. Performance Improvement of Colored Optical Packet Switching Thanks to Time Slot Sharing. Proceedings of the 14th Advanced International Conference on Telecommunications.

[21] Kamli A. (2020). Analysis and Optimisation of a New Futuristic Optical Network Architecture. Ecole Doctorale n = 580 Sciences et Technologies de l’Information et de Communication (STIC). Ph.D. Thesis.

[22] Domańska J., Kotuliak I., Atmaca T., Czachórski T. Optical Packet Filling. Proceedings of the 10th Polish Teletraffic Symposium PSRT2003.

[23] Mountrouidou X., Perros H.G. (2006). Characterization of the Burst Aggregation Process in Optical Burst Switching. Networking 2006: Networking Technologies, Services, and Protocols; Performance of Computer and Communication Networks; Mobile and Wireless Communications Systems.

[24] Li H., Thng I.L.J. (2007). Edge node buffer usage in optical burst switching networks. Photon Netw. Commun..

[25] Hernández J.A., Aracil J., López V., de Vergara J.L. (2007). On the analysis of burst-assembly delay in OBS networks and applications in delay-based service differentiation. Photon Netw. Commun..

[26] Toksöz M.A., Akar N. (2010). Dynamic threshold-based assembly algorithms for optical burst switching networks subject to burst rate constraints. Photon Netw. Commun..

[27] Kuaban G.S., Anyam E., Czachórski T., Rataj A. (2018). Performance of a Buffer Between Electronic and All-Optical Networks, Diffusion Approximation Model. ISCIS 2018: Computer and Information Sciences, Communications in Computer and Information Science.

[28] Czachórski T., Gelenbe E., Kuaban G.S., Marek D. Transient Behaviour of a Network Router. Proceedings of the 43th International Conference on Telecommunications and Signal Processing (TSP).

[29] Czachórski T., Gelenbe E., Kuaban G.S., Marek D. (2021). Time-Dependent Performance of a Multi-Hop Software Defined Network. Appl. Sci..

[30] Sivanathan A., Gharakheili H.H., Loi F., Radford A., Chamith Wijenayake A.V., Sivaraman V. (2019). Classifying IoT Devices in Smart Environments Using Network Traffic Characteristics. IEEE Trans. Mob. Comput..

[31] Atmaca T., Kamli A., Kuaban G.S., Czachórski T. (2021). Performance Evaluation of the Packet Aggregation Mechanism of an N-GREEN Metro Network Node. MASCOTS 2020: Modelling, Analysis, and Simulation of Computer and Telecommunication Systems, Lecture Notes in Computer Science.

[32] Gelenbe E. (1975). On Approximate Computer Systems Models. J. ACM.

[33] Gelenbe E. (1979). Probabilistic models of computer systems Part II: Diffusion approximations, waiting times and batch arrivals. Acta Inform..

[34] Ertürk M.A., Aydın M.A., Büyükakkaslar M.T. (2019). A Survey on LoRaWAN Architecture, Protocol and Technologies. Future Internet.

[35] Lavric A., Petrariu A.I., Popa V. (2019). Long Range SigFox Communication Protocol Scalability Analysis Under Large-Scale, High-Density Conditions. IEEE Access.

[36] Koike A., Ohba T., Ishibashi R. IoT Network Architecture Using Packet Aggregation and Disaggregation. Proceedings of the 2016 5th IIAI International Congress on Advanced Applied Informatics (IIAI-AAI).

[37] Homaei M.H., Salwana E., Shamshirband S. (2019). An Enhanced Distributed Data Aggregation Method in the Internet of Things. Sensors.

[38] Rezvanian A., Moradabadi B., Ghavipour M., Khomami M.M., Meybodi M.R. (2019). Introduction to Learning Automata Models. Learning Automata Approach for Social Networks. Studies in Computational Intelligence.

[39] Liu X., Zhao M., Liu A., Wong K.K.L. (2020). Adjusting forwarder nodes and duty cycle using packet aggregation routing for body sensor networks. Inf. Fusion.

[40] Bhandari S., Sharma S.K., Wang X. Latency Minimization in Wireless IoT Using Prioritized Channel Access and Data Aggregation. Proceedings of the GLOBECOM 2017 - 2017 IEEE Global Communications Conference.

[41] Mwakwata C.B., Malik H., Alam M.M., Moullec Y.L., Parand S., Mumtaz S. (2019). Narrowband Internet of Things (NB-IoT): From Physical (PHY) and Media Access Control (MAC) Layers Perspectives. Sensors.

[42] Liu J., Ansari N. The impact of the burst assembly interval on the OBS ingress traffic characteristics and system performance. Proceedings of the 2004 IEEE International Conference on Communications (IEEE Cat. No.04CH37577).

[43] Mountrouidou X., Perros H. (2020). On the departure process of burst aggregation algorithms in optical burst switching. Adv. Electron. Telecommun..

[44] Kuaban G.S., Czachórski T., Rataj A. (2018). A Queueing Model of the Edge Node in IP over All-Optical Networks. Computer Networks. CN 2018. Communications in Computer and Information Science.

[45] Bourguiba M., Haddadou K., Korbi I.E., Pujolle G. (2014). Improving Network I/O Virtualization for Cloud Computing. IEEE Trans. Parallel Distrib. Syst..

[46] Thakur S.R., Goudar R.M. (2014). Improving Network I/O Virtualization Performance. Int. J. Eng. Trends Technol. IJETT.

[47] Bourguiba M., Haddadou K., Pujolle G. (2012). Packet aggregation based network I/O virtualization for cloud computing. Comput. Commun..

[48] (2016). Center for Applied Internet Data Analysis (CAIDA) Based at the University of California’s San Diego Supercomputer Center February 2016 Data Sets, Equinix-Chicargo Link. https://data.caida.org/datasets/passive-2016/equinix-chicago/20160218-130000.UTC/.

[49] Garsva E., Paulauskas N., Grazulevicius G., Gulbinovic L. (2014). Packet Inter-arrival Time Distribution in Academic Computer Network. Elektron. IR Elektrotechnika.

[50] Cox R.P., Miller H.D. (1965). The Theory of Stochastic Processes.

[51] Kobayashi H. (1978). Modeling and Analysis: An Introduction to System Performance Evaluation Methodology.

[52] Liaw G.H., Wang S.Y., Kao T.L., Chen T.C. (2009). A Novel Packet Aggregation Mechanism for Enhancing VoIP Performance on IEEE 802.11 Wireless Mesh Networks. Int. J. New Technol. Res..

[53] Vishwanath A., Hinton K., Ayre R.W.A., Tucker R.S. (2014). Modeling Energy Consumption in High-Capacity Routers and Switches. IEEE J. Sel. Areas Commun..

[54] Hinton K., Jalali F., Matin A. (2015). Energy consumption modelling of optical networks. Photon Netw Commun..

[55] Ghiasian A. (2020). Impact of TCAM size on power efficiency in a network of OpenFlow switches. IET Netw..

[56] Sivaraman V., Vishwanath A., Zhao Z., Russell C. Profiling per-packet and per-byte energy consumption in the NetFPGA Gigabit router. Proceedings of the 2011 IEEE Conference on Computer Communications Workshops (INFOCOM WKSHPS).

[57] Tucker R., Hinton K. (2014). Energy-Efficient Networking; Computer Science, Corpus ID: 167877010. www.semanticscholar.org.

[58] Czachórski T., Gelenbe E., Suila K., Marek D. Time Dependent Diffusion Model for Security Driven Software Defined Networks. Proceedings of the Second InternationalWorkshop on Stochastic Modeling and Applied Research of Technology (SMARTY 2020).

[59] Frohlich P., Gelenbe E., Fiolka J., Checinski J., Nowak M., Filus Z. (2021). Smart SDN Management of Fog Services to Optimize QoS and Energy. Sensors.

